# Ecological restoration of habitats invaded by *Leucanthemum vulgare* that alters key ecosystem functions

**DOI:** 10.1371/journal.pone.0246665

**Published:** 2021-03-26

**Authors:** Mohd Asgar Khan, Khursheed Hussain, Manzoor A. Shah

**Affiliations:** Department of Botany, University of Kashmir, Srinagar, Jammu & Kashmir, India; Feroze Gandhi Degree College, INDIA

## Abstract

Precise assessment of the impacts of invasive alien species (IAS) on ecosystem structure and functions is paramount for implementing appropriate management and restoration strategies. Here we investigated the impacts of *Leucanthemum vulgare* (ox-eye daisy), an aggressive invader in Kashmir Himalaya, on species diversity and primary productivity. We also evaluated bunch of strategies for the ecological restoration of the habitats invaded by this species. We found that uninvaded plots harbored on an average of 6.11 (±2.92) more species per 1m^2^ of quadrat than invaded plots. At multivariate scale, the ordination (nMDS) and ANOSIM exhibited significant differences between invaded and uninvaded plots with R = 0.7889 and *p <* 0.001. The decrease in diversity indices in invaded as compared to uninvaded plots was associated with more productive plant communities due to *Leucanthemum* invasion. Higher altitude Gulmarg site was more affected by *Leucanthemum* invasion than lower altitude Drung site. We tested different approaches for restoration and management of invaded habitats that include herbicide treatment at seedling stage, herbicide treatment before and after flowering stage, mowing and herbicide treatment together, joint mowing, digging and herbicide treatment and *Leucanthemum* uprooting. Among these treatments, uprooting and combined digging, mowing and herbicide treatment proved to be most effective in controlling *Leucanthemum* invasion. The implications of these results for effective management of ecologically sensitive and socio-culturally important landscapes are discussed.

## Introduction

The growing anthropogenic pressures on natural ecosystems that drive immense biodiversity loss globally, hamper the functioning of ecosystems and their ability to provide essential services [1, 2] characterize the Anthropocene. Nearly one-sixth of the global land area, including significant areas of developing economies and global biodiversity hotspots are currently highly vulnerable to invasion by alien species [3]. Rapidly spreading invasive alien species (IAS) seriously threaten the global environment in view of multitude of undesirable impacts on invaded communities, especially by displacing native species [4–8]. The IAS have been not only linked to biodiversity loss [9–11] but also to changes in ecosystem functions, including nutrient and carbon regulation [12, 13], primary productivity [14] and soil microbial processes [15]. It is a well-established fact that the plant species richness and functional diversity increases local net primary productivity, principally through more exploitation of resources, or niche complementarity [16]. However, contrary to the diversity-productivity experiments, alien plant invasions create a paradox by increasing the local net primary productivity [13, 17] and reduces native plant diversity in the invaded communities, often to the extent of becoming like monotonous stands [18]. Rather than depleting soil resources as productivity increases, invasions often increase soil stocks, pools and fluxes of nitrogen through processes regulated by microbial communities [19]. A meta-analysis of 94 studies revealed that the average increase in annual net primary productivity was over 80% in invaded ecosystems [13].

The United Nations has declared 2021–2030 as the decade of ecological restoration. Taking cue from this, it is imperative to undertake effective restoration and management strategies for invaded habitats, especially which are ecologically fragile and socio-culturally important. Globally multi-scale programs (local, regional, national, and international) are in place to reduce existing and potential future impacts caused by IAS. The approaches range from low impact practices, involving only removal of invasive species by myriad of manipulative treatments aimed at reducing the presence, abundance, or impacts of invasive species and favoring native species, to massive exercises of reintroducing native species [20]. Many of such efforts have been successful in mitigating the negative impacts of IAS and restoring degraded ecosystems [20].

The valley of Kashmir in the Himalayan biodiversity hotspot harbors rich biodiversity and invaluable bio-resources, including incredible plant wealth [21]. However, this bio-wealth faces myriad of threats, of which invasion by alien species is one of the most severe ones in recent times. Although many mountains are considered relatively immune to invasions compared to surrounding lowland ecosystems [22], primarily because only a few of the potential invaders from the lowlands manage to spread along steep climate gradients to high elevations [23].

In Kashmir Himalaya, *Leucanthemum vulgare* (ox-eye daisy) is one of the alien species that has caused environmental damage in mountain habitats. *Leucanthemum vulgare*, introduced during the British era in the valley as an ornamental plant for its beautiful white blooms in the recreation gardens and tourist spots, is now growing profusely in the wild amid shift from cultivation [24]. Though in Kashmir valley at present it shows constrained distribution in the peripheries of tourist spots, meadows and forest openings [24], it is recognized globally as high-altitudinal invasive species and places endemic diversity and life-supporting ecological services in these regions at high risk [24, 25]. Other researchers, [2, 26] also reported *L*. *vulgare* as one of the worst IAS in the protected areas in the world. Recently it has stretched its reach, threatening a range of rare plant species listed under the Biodiversity Conservation Act 2016 (Saving Our Species Database, accessed 18 April 2016) [27].

Despite being a global invader, *L*. *vulgare* has not attracted the attention it deserves for its scientific management except for few studies [24, 25, 28]. More importantly, there has hardly been any credible attempt for its ecological restoration of habitats invaded by *L*. *vulgare* [5]. Therefore, we deemed it imperative to understand the impacts of this global invader on the structure and functioning of the invaded communities, and formulate and implement effective invasion management strategies through ecological restoration of invaded habitats in otherwise immaculate landscapes such as Gulmarg in Kashmir. To date, management of *L*. *vulgare* has been mostly relied upon chemical and mechanical approaches with rather erratic success. Hand pulling, grubbing, and hoeing have been proved effective in controlling small, isolated populations of *L*. *vulgare* [29].

In this backdrop we asked two fundamental questions in the present study: (a) what is the impact of *L*. *vulgare* on species diversity and ecosystem functioning with particular reference to net primary productivity? (b) which strategies are most effective for ecological restoration of the habitats invaded by this species?

## Methodology

### Study species

The focal species for the present study was *Leucanthemum vulgare*, a diploid species native to Europe and western Asia. It is a perennial forb belonging to family Asteraceae that primarily reproduces by seeds, although rhizomes also contribute to its propagation. The plant is a prolific seed producer and a single, healthy, robust plant may produce up to 26,000 seeds. Germination occurs throughout the growing season, and new seedlings appear in autumn quickly after seed dispersal, but most new seedlings emerge in the spring when conditions are favorable.

### Study area

The study was carried out in a high-altitude mountainous region of the valley of Kashmir called Gulmarg (2600 m asl) with a picturesque landscape comprising of coniferous forests dominated by Blue Pine (*Pinus wallichaina*) and alpine meadows. The climate is predominantly temperate type with wet and cold winters and relatively dry and hot summers. The average temperature during summer is 17.6 ^o^C and winter is—4.4 ^o^C. The target species chosen for the present study (*L*. *vulgare*) together with some other alien species have taken over a significant part of the Gulmarg landscape forming apparently monotonous stands thereby affecting the scenic view of this famous tourist spot, besides inducing huge ecological problems.

### Species diversity assessment

**Fig 1:**
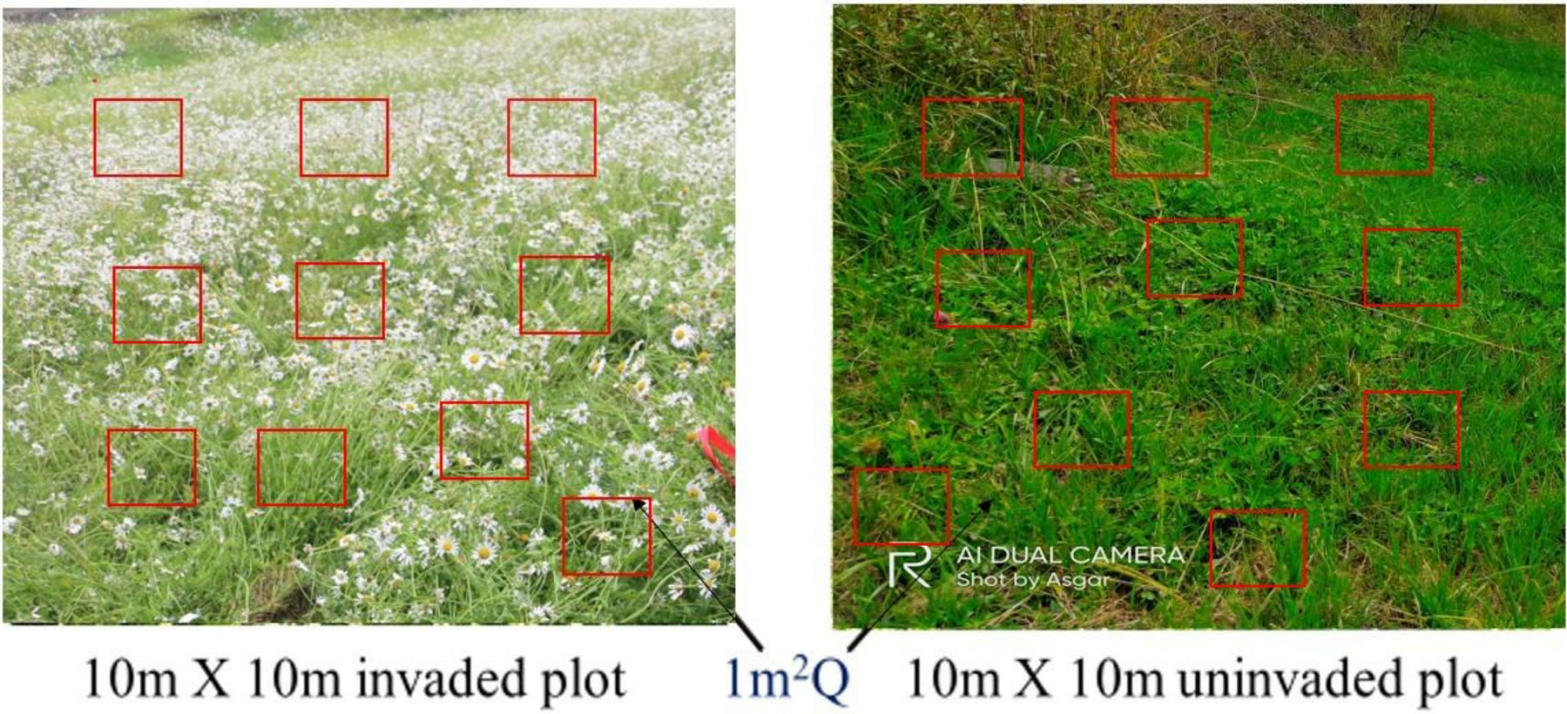
Design of the experimental plots showing 1m^2^ quadrats in 10m x 10 m plots.

We identified 10m x 10m plots (Fig 1) with at least 50% cover of *L*. *vulgare* (hereafter referred as the invaded plot) adjacent to a plot of the same size with no *L*. *vulgare* (uninvaded plot), as determined by visual estimation. Both the invaded and uninvaded plots were matched for elevation, aspect, slope, and landscape position to minimize difference in confounding environmental factors. The invaded and uninvaded plots were separated by a 5m to 15m buffer strip [5]. In each plot 10 1m^2^ quadrats (Fig 1) were randomly laid down, surveyed and sampled for vegetation in mid-June, mid-August, and mid-October during the years 2018 and 2019. All the herbaceous vascular plant species, seedlings of shrubs and tree species rooted within each quadrat were noted. The percentage of *L*. *vulgare* cover, co-occurring species cover, litter cover, bare ground percentage, individual count and height was monitored. Species were identified with the help of standard taxonomic literature [30, 31] followed by the expert consultation.

### Studies on primary productivity

For studying the impact of *L*. *vulgare* on net primary productivity same experimental settings were used as for diversity analysis. We estimated aboveground and belowground net primary productivity at the target sites by measuring peak standing above- and below-ground biomass, respectively in both invaded and uninvaded plots. We harvested all plant biomass produced in the current year within each 0.25 m^2^ plot by clipping at ground level [14]. The biomass was oven dried at 72°C for 72 hours and weighed. Belowground biomass was measured by soil core method [32]. Plots were selected and marked at the center for sampling below ground biomass. 5–10 cm soil core were drilled into the soil and soil was collected along with the roots. Roots were separated and thoroughly washed with tap water. Roots collected were oven dried for the estimation of dry weight at 70˚C to a constant weight for 8 hours. Dry weight of roots was estimated for the volume of core sampler (calculated from its diameter and depth) for all sampled plots. Root biomass was then extrapolated to per plot basis using dry weight of the root samples.

### Restoration of *L*. *vulgare* invaded plots

A field experiment was conducted for two years from April 2018 to June 2020 at the Gulmarg landscape infested heavily by the *L*. *vulgare*. Study plots were matched for elevation, aspect, slope, and landscape position to minimize difference in environmental factors. The experiment consisted of five different 5x10m plots and six different treatments as detailed below. In each plot, for each treatment we laid five 1-m^2^ subplots (replicates) in a split plot design. In addition, we located 5 permanent 1-m^2^ plots each of invaded and uninvaded ones in the same study site, as a possible reference for comparing with before and after treatment plots. In 2018, before the experimental treatments, we estimated percent cover for each species within 1-m^2^ vegetation plots using a modified six-class Braun-Blanquet scale (<2%, 2–5%, 6–25%, 26–50%, 51–75%, 76–100%) in all treatment plots. We also estimated the species richness, evenness and diversity of each plot. Same parameters were estimated post treatment in each plot as were done before treatment. The treatments applied were herbicide treatment at seedling stage, herbicide application before and after flowering, mowing and herbicide treatment together, mowing, digging and herbicide treatment jointly, and *Leucanthemum* uprooting. For the *Leucanthemum* uprooting treatment, all *L*. *vulgare* individuals were hand pulled twice a year and placed outside of the plots while avoiding other species. Hand-pulling was used because it is a common control method for many invasive plants, does not require equipment, is relatively low-cost, and is non-chemical. The seedling stage *L*. *vulgare* recommended herbicide treatment was 2, 4 D ester in combination with Dicamba (0.7% + 0.5% NIS) 2 to 4 pts per Acre. This herbicide is known to be effective at seedling stage to rosette stage. The before flowering herbicide treatment was 2–2.5 pts per acre of Aminopyralid in combination with 2, 4 D (GrazoNext Forefront R&P). This treatment was applied in the spring followed by the application of herbicide after flowering treatment which was 0.5 to 1 oz 1gram/gallon of Metasulfuron methyl (Escort). The herbicide treatment at seedling stage was tested in addition to post-emergent herbicide treatments because *L*. *vulgare* produce abundant seed that can remain viable in the seed bank up to 6 years [33], and this treatment was designed to kill seeds as they germinate. Herbicides were applied with backpack sprayers. Other treatments include combination of chemical and mechanical methods, in one treatment we did the mowing followed by digging and then herbicide treatment to the same plot and in other we did mow followed by herbicide treatment. In mowing, all plants present in the plot were clipped from the base and placed outside the plot. Herbicide treatment at seedling stage was done in the 2^nd^ week of April 2018. At the time of treatment, *L*. *vulgare* seeds had germinated and seedlings were approximately 5–15 cm in height. Hand-weeding (*L*. *vulgare* uprooting), mowing, digging and before flowering herbicide treatments were conducted in the 1^st^ week of June 2018. Herbicide after flowering treatment was given in the last week of July when the Leucanthemum vulgare is in full bloom and our intension was not to let the plant to mature its seeds. Same treatments were applied likewise in the 2^nd^ year. Overall, this experimental design included six treatments applied across three growing seasons and a wide range of invaded plots, and uninvaded plots as reference for restoring habitats invaded by *L*. *vulgare*.

### Data analyses

Impact of *L*. *vulgare* invasion on species diversity were evaluated by calculating and comparing diversity indices including Shannon–Weaver index of diversity (H´), Margalef’s index of richness (R), Simpson index of dominance (λ) and index of evenness (J’) for invaded and uninvaded plots at all sampling sites [34]. To ensure that our sampling at all sites was adequate and robust, Rarefaction curves were plotted using SPSS software.

For invasion impact analysis, diversity indices including species richness (R), species evenness (J’), Shannon index of diversity (H′) and Simpson index of dominance (λ) were calculated for invaded as well as for uninvaded plots. The analysis of variance (ANOVA) of these diversity indices was conducted with invasion status and sites as factors using IBM SPSS v. 21 [35].

We used non-metric multidimensional scaling analysis (NMDS) to assess the potential impact of *L*. *vulgare* on species composition. Abundance data pooled across sites was subjected to NMDS based on Bray-Curtis dissimilarity matrix to account for variation in the species composition by invasion status (invaded vs uninvaded). Prior to NMDS, we excluded the rare species that occurred in only one or two quadrats from subsequent analysis [36, 37]. Also, we excluded *L*. *vulgare* from the analysis, as our aim was to examine the variations in the species composition as a result of invasion by *L*. *vulgare*. The range of clustering of sites and plots in response to invasion were assessed by analysis of similarity (ANOSIM) and similarity percentage (SIMPER). ANOSIM relates mean difference of ranks between and within groups, generating the Global statistic (R). Since the values of Global statistic (R) range from -1 to +1, values approaching 0 and negative values indicate similarity among groups and values approaching +1 indicate a strong dissimilarity among groups [38, 39]. SIMPER identified species contributed most to average dissimilarity between groups (invaded and uninvaded plots). This technique calculates average impact of each species contributing to dissimilarity between groups [40–42]. Values of percentage similarity between groups range from 0 to 100, with 100 stating maximum similarity. Therefore, to estimate the amount of variation in the community composition and to identify species contributing to those differences we performed similarity percentage test (SIMPER) [41, 42]. The analysis was carried using the PAST software.

For invasion impact analysis, above and belowground net primary productivity were calculated for uninvaded as well as for invaded plots. The above values were subjected to analysis of variance (ANOVA) to compare difference in above and belowground productivity with invasion status and sites. Linear regression was carried out to find the relation between species richness and net primary productivity under the scenario of plant invasion.

To understand the effectiveness of different treatment approaches for *L*. *vulgare* control, we compared the reference plots with the treatment plots before and after 2 years of treatments, in terms of response variables like *L*. *vulgare* cover percentage, co-occurring species cover percentage, species richness, species evenness and Shannon diversity index (H). We used ANOVA, to find the differences in these response variables between reference plots and the treatment plots. This will allow us to find the best treatment approach that shows the significant differences from the reference plots.

## Results

### Impact on species diversity

**Fig 2:**
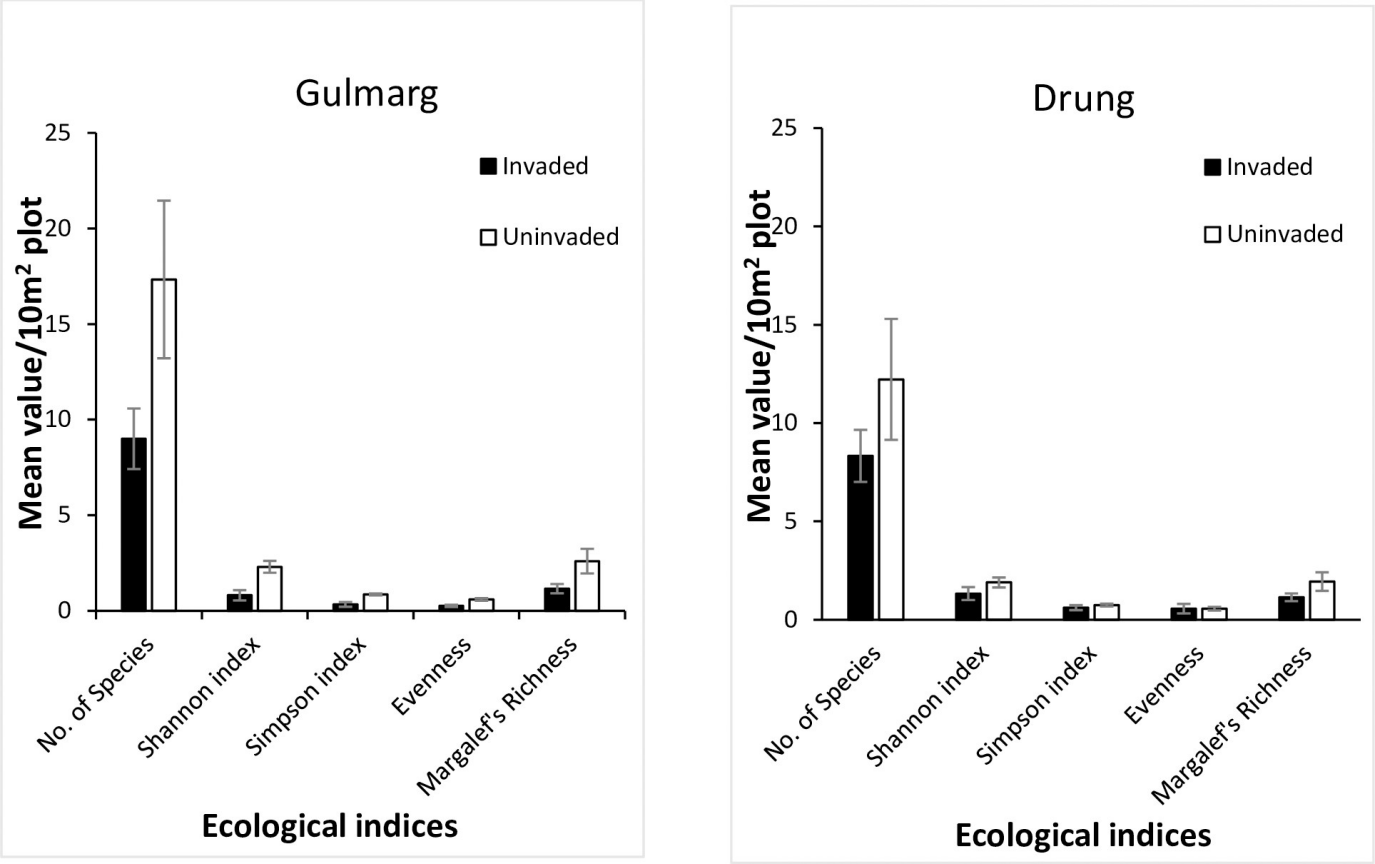
Mean values/10m^2^ for ecological indices of invaded vs. control plots in different sites.

A total of 67 plant species from 55 genera were documented during the study. Of these, a total of 59 species were recorded in uninvaded plots compared to 45 in species in *Leucanthemum* infested plots. Mean species diversity and richness was higher in un-invaded than invaded plots (Fig 2). This was corroborated by comparison of ecological indices reflecting significant difference between invaded and uninvaded plots (Table 1). However, the impact of *L*. *vulgare* invasion didn’t vary much between the two study sites. Invaded plots harbored on an average 8.66±1.45 (mean ± SD, n = 10) species per m^2^ as against 14.77±4.39 species per m^2^ in uninvaded plots. So uninvaded plots harbored 6.11±2.92 more species than invaded plots and the difference was statistically significant (t = -5.59, df = 20.67, *p* = 0.004). The number of species per plot also varied between sites with a significant difference (f = 9.78, *p* = 0.004). Uninvaded plots as compared to invaded plots exhibited higher values of Shannon index of diversity by a difference of 1.03±0.365, and the difference was significant (t = -8.32, df = 33.43, *p* = 0.001). Similarly, uninvaded plots also exhibited higher values of Simpson index of dominance by a difference of 0.33 ± 0.125; species evenness by 0.16± 0.15; and species richness by 0.76±0.425 (Table 2). For individual sites, *L*. *vulgare* invasion had significant impacts on diversity indices except species evenness at site 2, i.e., Drung (Table 2).

**Table 1:** Analysis of variance (ANOVA) of invasion impacts and sites on diversity indices of local plant communities.

Ecological index		SUMMARY ANOVA		Mean(±SD)	
	Sites	Invasion status (IS)	SitesˣIS interaction	Uninvaded (30)	Invaded (30)
Species No. (S)	**	***	**	8.66±1.45	14.77±4.39
Shannon index of diversity (Hꞌ)	NS	***	***	1.07±0.39	2.10±0.34
Simpson index of dominance (λ)	*	***	***	0.47±0.18	0.80±0.07
Species evenness (Jꞌ)	**	***	***	0.41±0.23	0.57±0.07
Margalef Richness (R)	NS	***	***	1.51±0.21	2.27±0.64

±SD indicates ‘standard deviation’

***p ≤ 0.001

** *p* ≤ 0.02

* *p* ≤ 0.05; NS (not significant) *p* > 0.05.

**Table 2:** Student’s t-test for significance of differences between uninvaded and invaded plots at different sites.

Sites	Number of species (S)	Shannon index of diversity (Hꞌ)	Simpson index of dominance (λ)	Species evenness (Jꞌ)	Margalef Richness (R)
Gulmarg	***	***	***	***	***
Drung	**	***	**	NS	***

*** *p* ≤ 0.001

** *p* ≤ 0.02

* *p* ≤ 0.05; NS (not significant) *p* > 0.05.

### Impact on community composition

**Fig 3:**
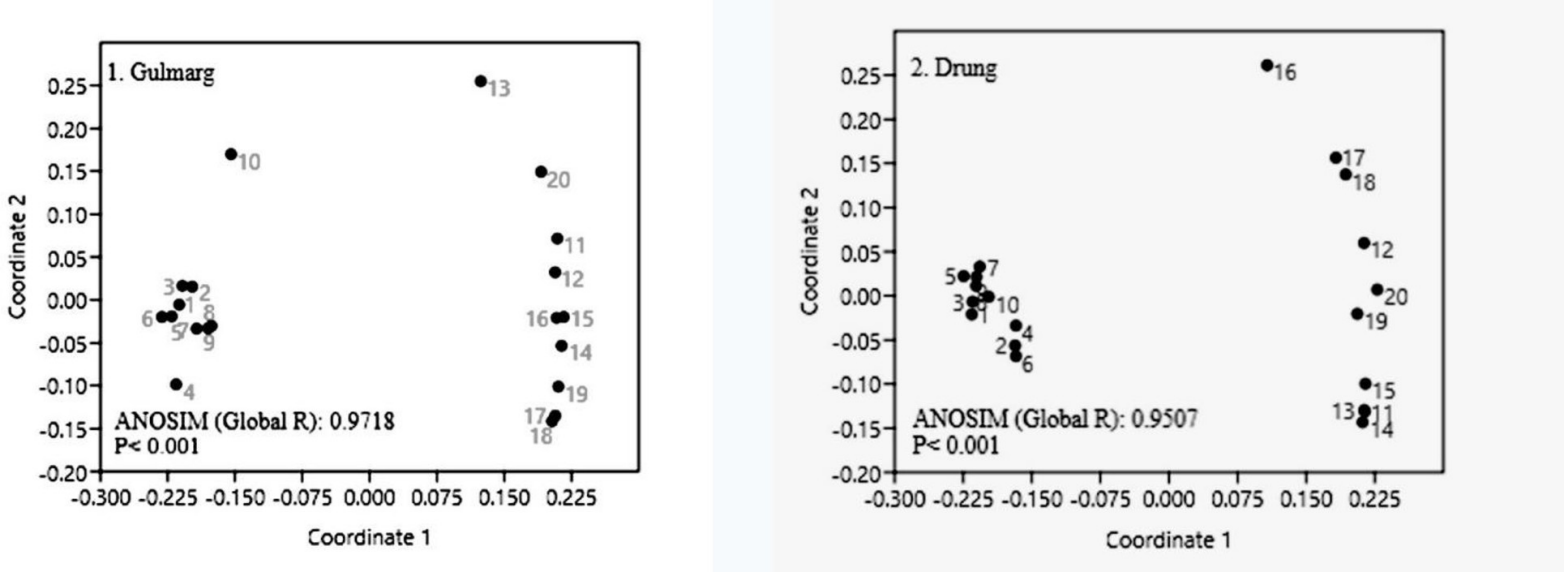
Multidimensional scaling (MDS) and analyses of similarity (ANOSIM) for two study sites, Gulmarg (left) and Drang (right).

**Fig 4:**
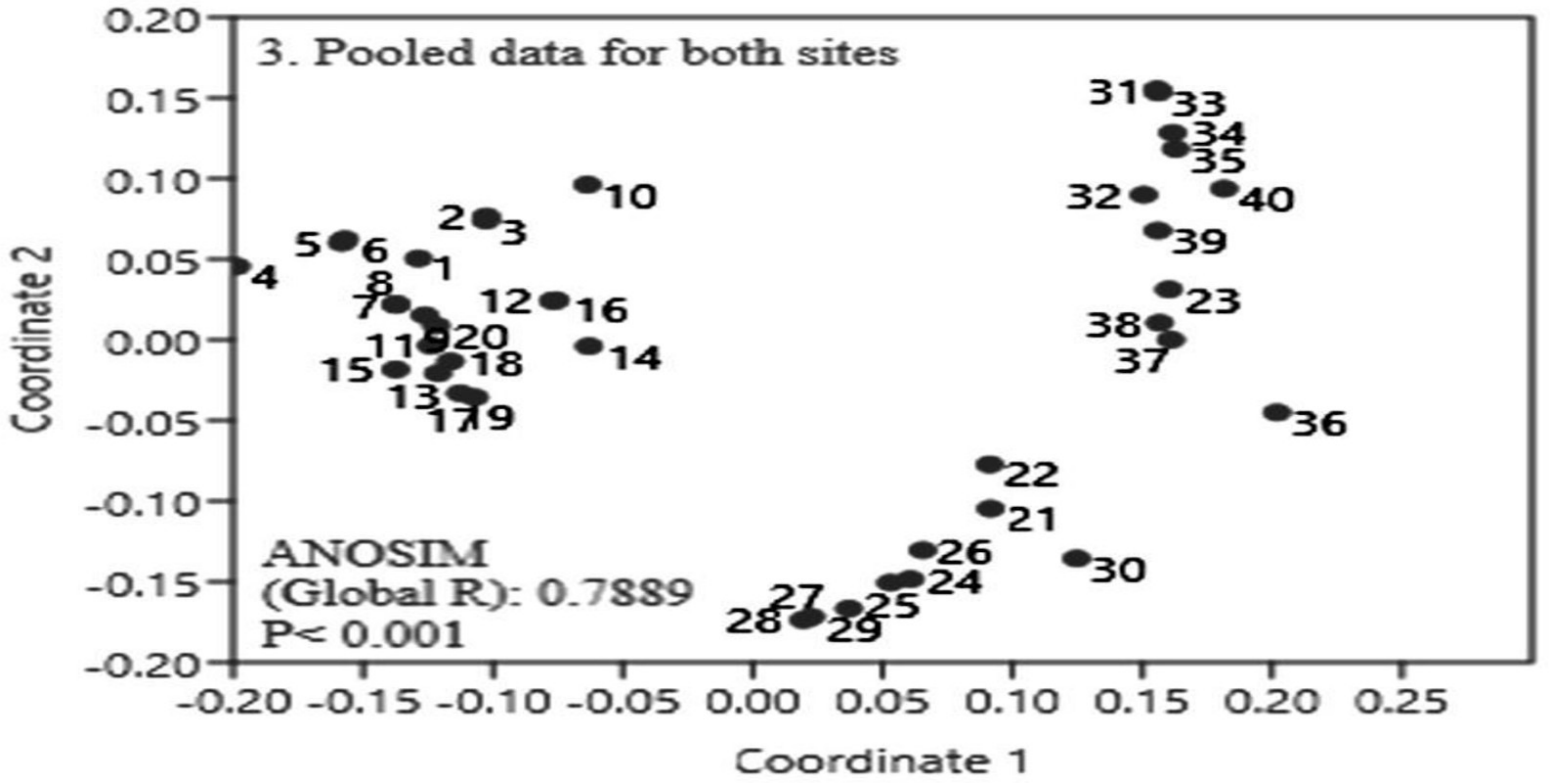
Multidimensional scaling (MDS) and analyses of similarity (ANOSIM) for pooled data of both sites (1–20 indicate invaded plots, 21–40 control ones).

The ordination (nMDS) and ANOSIM showed significant differences between species composition of invaded and uninvaded plots in both sites with global R values of 0.9718 (*p* = 0.0002) for Gulmarg and 0.9507 (*p* = 0.0002) for Drung (Figs 3 and 4). The dissimilarity between invaded and uninvaded plots was noticed slightly more at Gulmarg site. Similarity percentage (SIMPER) analysis of data suggested species contributing most to average dissimilarity between uninvaded and invaded groups. This analysis also computed average contribution of species causing dissimilarity. SIMPER analysis showed 76.74% overall dissimilarity among invaded and uninvaded plots (Table 3). Few worth mentioning species out of list given in Table 3 that distinguish invaded and un-invaded plots include *Poa annua*, *Cynodon dactylon*, *Poa angustifolia*, *Sibbaldia cuneata*, *Carex* spp., *Poa pratensis*, *Trifolium repens*, *T*. *pratense*, *Impatiens thomsonii*, *Silene coronaria*, *Ranunculus luteus*, *Bromus japonicus*, *Geranium nepalense*, *Fragaria nubicola*, *Impatiens brachycentra*, *Urtica dioica*, *Digitalis grandiflora* and *Rumex acetosa*.

**Table 3:** SIPMER analysis of *Leucanthemum* invaded and uninvaded sites in Gulmarg and Drung.

Average dissimilarity = 76.74%					
Species	Average Dissimilarity	Average Contribution	Cumulative sum	Average abundance IN	Average abundance UN
*Poa annua*	11.83	15.41	15.41	89.2	61.6
*Cynodon dactylon*	10.77	14.04	29.45	38.2	71.1
*Poa angustifolia*	6.587	8.584	38.04	8.8	45.4
*Sibbaldia cuneata*	4.628	6.031	44.07	7.48	36.3
*Carex* spp.	3.405	4.437	48.51	2.54	26.5
*Poa pratensis*	3.398	4.428	52.93	28.7	0
*Trifolium repens*	3.338	4.349	57.28	27.4	33
*Impatiens thomsonii*	3.174	4.137	61.42	3.1	1.06
*Silene coronaria*	2.223	2.897	64.32	0	18.9
*Ranunculus laetus*	2.01	2.619	66.94	10.1	9.64
*Trifolium pratense*	1.921	2.504	69.44	14.8	13.8
*Bromus japonicus*	1.517	1.977	71.42	5.31	7.65
*Geranium nepalense*	1.489	1.94	73.36	1.7	10
*Fragaria nubicola*	1.356	1.767	75.12	31.12	9.59
*Impatiens brachycentra*	1.343	1.75	76.87	5.36	6.56
*Urtica dioica*	1.249	1.627	78.5	0.383	12.1
*Digitalis grandiflora*	1.036	1.35	79.85	1.48	7.35
*Rumex acetosa*	0.9267	1.208	81.06	3	4.19
*Unknown 1*	0.8934	1.164	82.22	0.793	6.6
*Prunella vulgaris*	0.7599	0.9902	83.21	1.14	5.55
*Geum elatum*	0.7493	0.9765	84.19	0	4.58
*Galium aparine*	0.7412	0.9659	85.15	0.948	4.46
*Pedicularis pectinata*	0.7222	0.9412	86.1	0.2	5.24
*Oxalis corniculate*	0.6879	0.8965	86.99	0	4.13
*Plantago Lanceolata*	0.6821	0.8889	87.88	2.46	3.83
*Stellaria media*	0.5355	0.6978	88.58	1.26	3.41
*Rumex nepalensis*	0.4707	0.6133	89.19	0.378	3.18
*Epilobium laxum*	0.4301	0.5605	89.75	0	2.5
*Viola odorata*	0.4198	0.547	90.3	0.314	3.33
*Digitalis purpurea*	0.4104	0.5349	90.83	0	2.77
*Persicaria amplexicaulis*	0.3959	0.516	91.35	2.18	1.17
*Chenopodium album*	0.3853	0.5021	91.85	0.472	2.03
*Achillea millefolium*	0.3753	0.4891	92.34	0	3
*Myosotis arvensis*	0.3734	0.4866	92.83	2.92	0
*Epilobium royleanum*	0.3516	0.4582	93.29	0	2.25
*Plantago major*	0.3512	0.4576	93.74	0	2.77
*Mentha* spp.	0.3474	0.4527	94.2	1.44	1.34
*Nepeta cataria*	0.3471	0.4523	94.65	1.58	1.25
*Chrysopogon fulvus*	0.3114	0.4058	95.05	2	0.6
*Taraxacum officinale*	0.2969	0.387	95.44	0	2.21
*Unknown 2*	0.2709	0.3531	95.79	0.35	1.76
*Veronica persica*	0.257	0.3349	96.13	0	1.69
*Rumex hastatus*	0.2539	0.3309	96.46	0.506	1.59
*Cirsium arvense*	0.2178	0.2838	96.74	1.17	0.642
*Geranium sibiricum*	0.2167	0.2824	97.03	0.25	1.41
*Polygonum amplexicaule*	0.2006	0.2615	97.29	0.05	1.51
*Indigofera*	0.1777	0.2316	97.52	0	1.59
*Picrorhiza kurroa*	0.1773	0.231	97.75	0	1.57
*Lamium album*	0.1599	0.2083	97.96	0.986	0
*Viola biflora*	0.1552	0.2023	98.16	0	1.23
*Achyranthes aspera*	0.1547	0.2016	98.36	1.04	0
*Unknown*	0.1432	0.1866	98.55	0.575	0.6
*Cirsium vulgare*	0.1187	0.1547	98.7	0.25	0.786
*Corydalis rutifolia*	0.1175	0.1531	98.86	0	0.78
*Linaria dalmatica*	0.1126	0.1467	99	0	0.821
*Euphorbia cornigera*	0.09901	0.129	99.13	0	0.733
*Bergenia ciliata*	0.09608	0.1252	99.26	0.36	0.45
*Sambucus wightiana*	0.08122	0.1058	99.36	0.45	0
*Capsella bursa-pastoris*	0.07842	0.1022	99.47	0.622	0
*Cirsium falconeri*	0.07591	0.09893	99.57	0.553	0
*Alliaria petiolate*	0.07486	0.09755	99.66	0	0.688
*Clinopodium*	0.0617	0.0804	99.74	0	0.431
*Umbrosum*					
*Dryopteris* sp.	0.05307	0.06916	99.81	0.38	0
*Isodon rugosus*	0.03862	0.05033	99.86	0	0.35
*Viburnum cotinifolium*	0.03638	0.0474	99.91	0	0.335
*Ajuga parviflora*	0.03498	0.04558	99.96	0	0.221
*Phytolacca acinosa*	0.03404	0.04436	100	0	0.271

### Impact on net primary productivity

**Fig 5:**
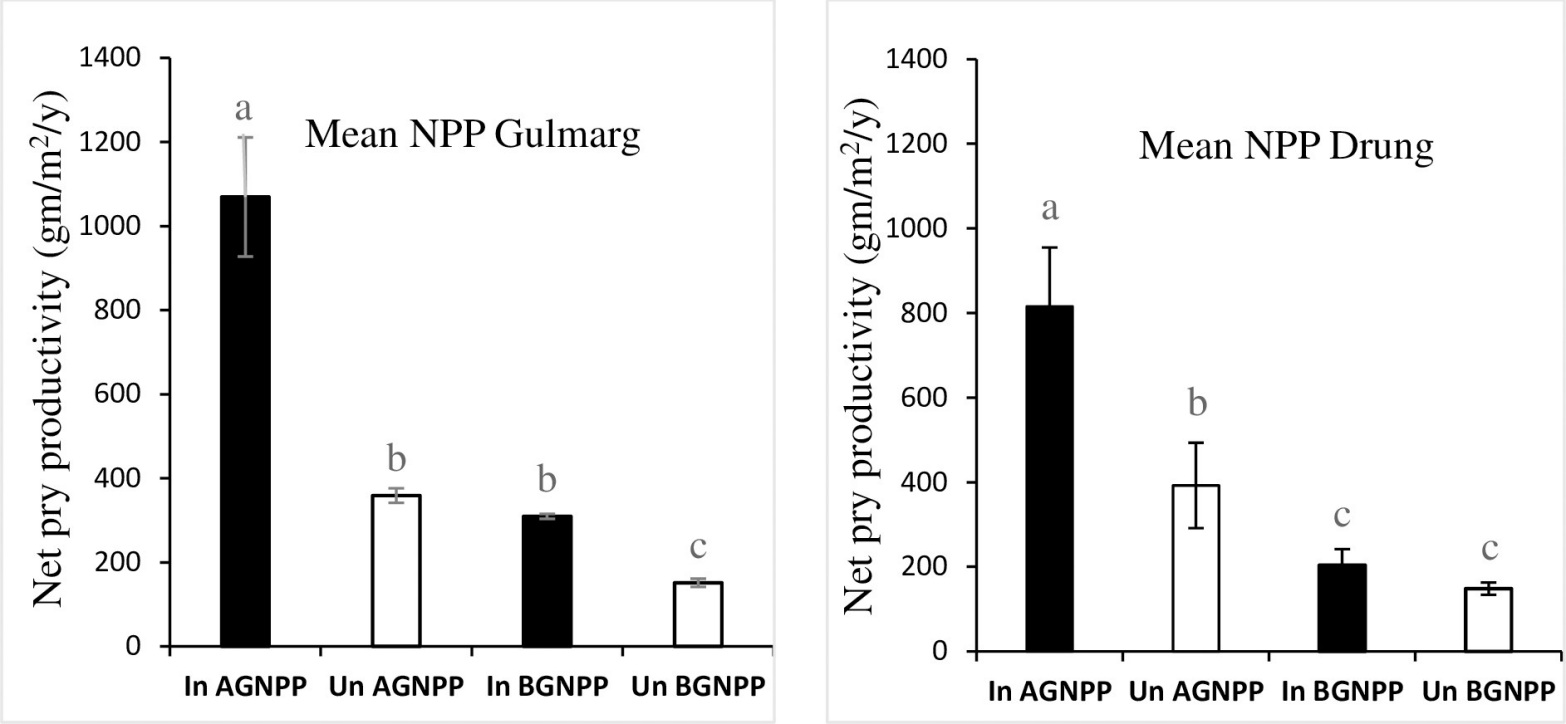
Mean value and comparison of above and belowground Net pr. productivity (g/m^2^/y) between Gulmarg and Drung.

On an average, invaded plots produced higher aboveground net primary productivity (AGNPP) and belowground net primary productivity (BGNPP) than nearby un-invaded plots. Both sites, Gulmarg and Drung, were found to be consistent in producing higher AGNPP and BGNNP in invaded plots (Fig 5). In Gulmarg invaded patches produced 181.08% more aboveground biomass than invaded plots (F = 256.88, df = 3, *p* <0.001) while as in case of Drung invaded plots produced 123.04% more aboveground biomass than uninvaded plots (F = 179.2777, df = 3, *p* < 0.001).

### Diversity-productivity relationship

**Fig 6:**
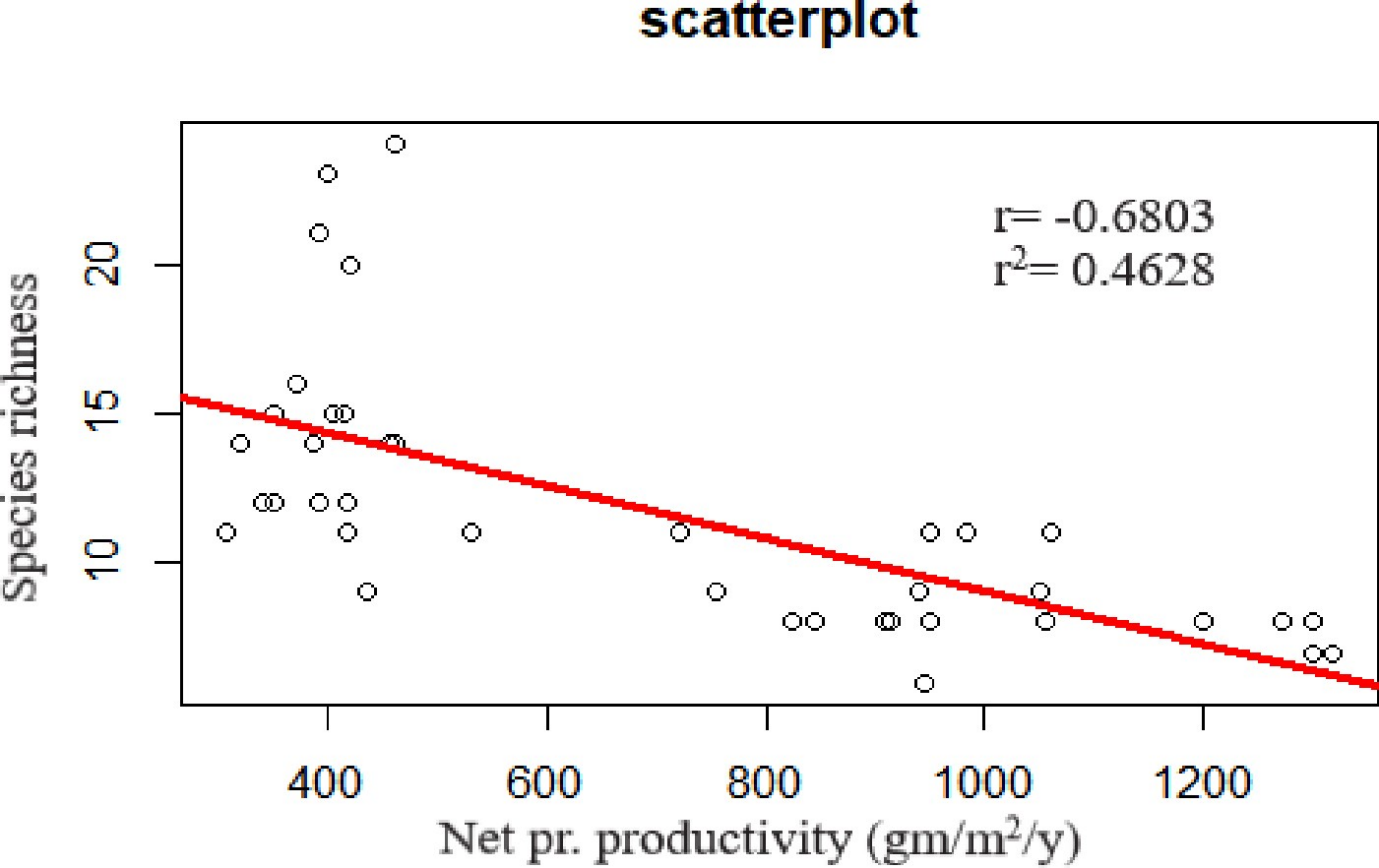
Scatterplot showing the negative correlation between net primary productivity and species richness.

AGNPP was negatively correlated with species richness (Fig 6; F = 32.74, df. = 38, *p* < 0.001, R ^2^ = 0.4628). Linear regression model clearly indicates that there is decreased species richness but increased net primary productivity in invaded plots, and *vice versa* in uninvaded plots (Fig 6). Analysis was done using R studio Version 1.1.463.

### Ecological restoration and management

**Fig 7:**
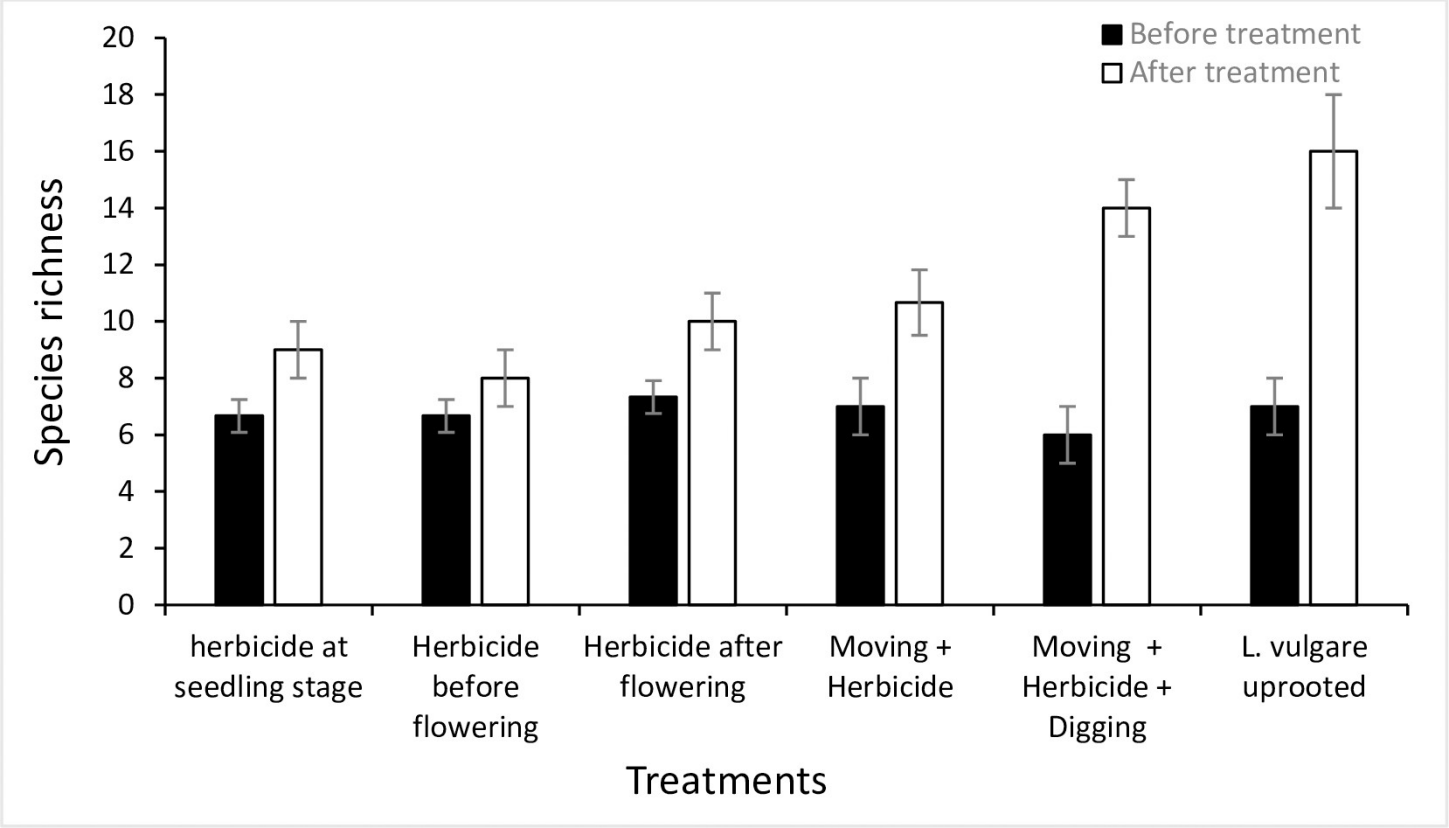
Species richness (per 1 m^2^ plot) before and after treatment for each treatment plot. *L*. *vulgare* treatment showing highest species richness after treatment with a significant difference when compared with reference invaded plot (*p* = 0.0001).

**Fig 8:**
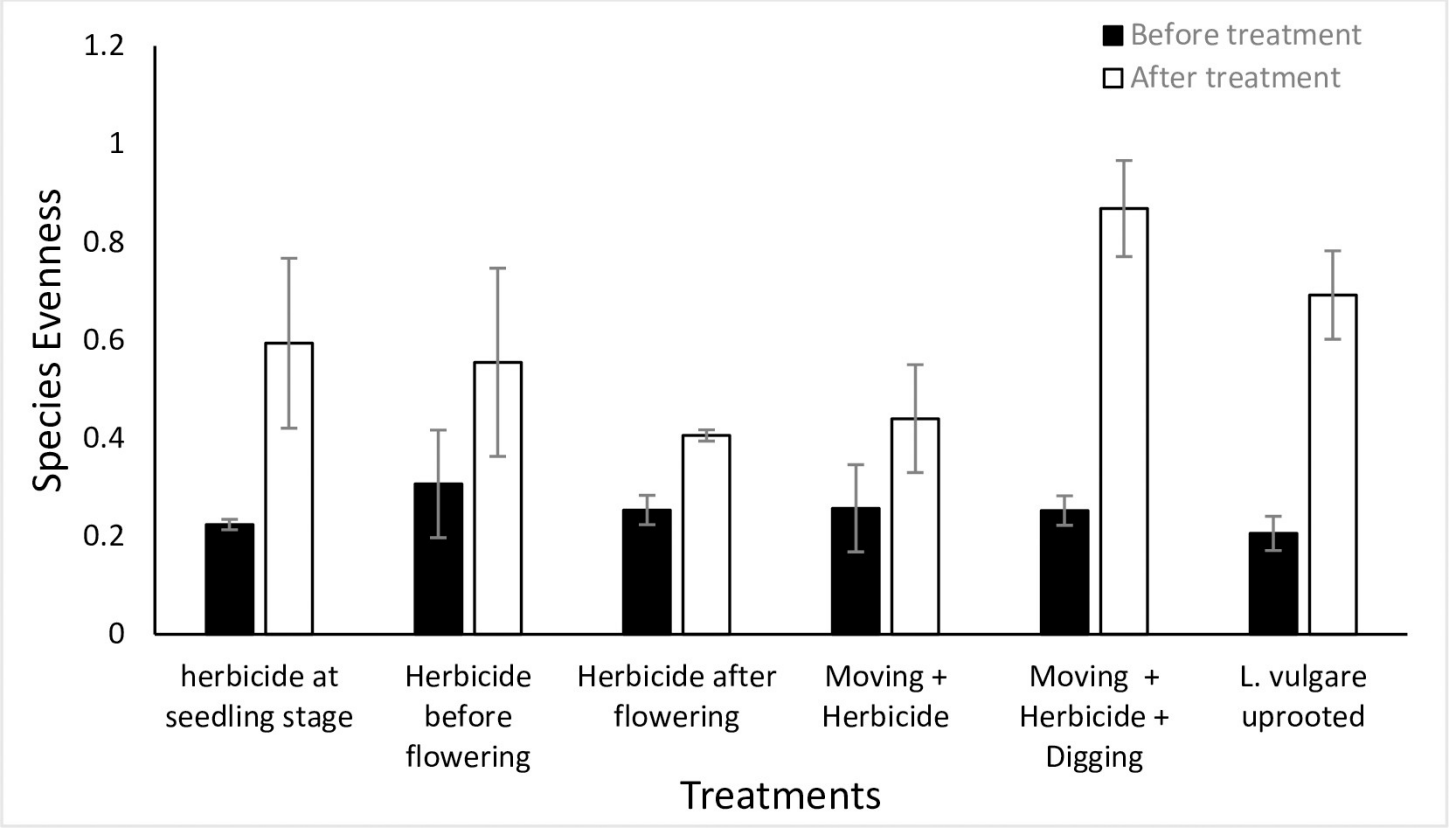
Species evenness (per 1 m^2^ plot) before and after treatment for each treatment plot with Mowing+herbicide+digging treatment showing highest species evenness after treatment with a significant difference when compared with reference invaded plot (*p* = 0.0001).

**Fig 9:**
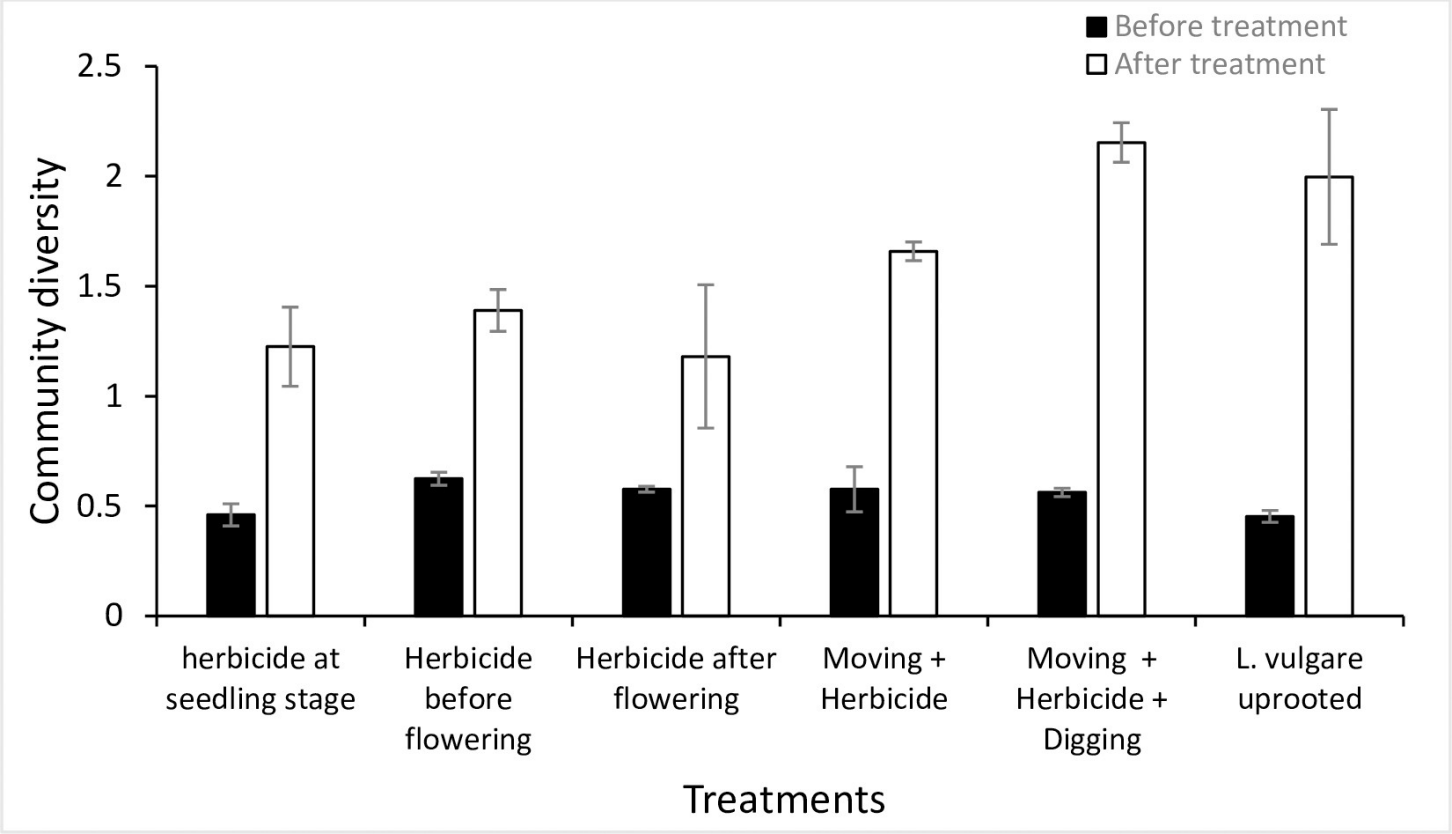
Community diversity (per 1 m^2^ plot) before and after treatment for each treatment plot with mowing + herbicide + digging treatment showing highest species diversity after treatment with a significant difference when compared with reference invaded plot (*p* = 0.0001).

Before treatments, as expected in a randomized experiment, treatment plots and invaded reference plots were not different from each other. However, the data collected during two years of annual treatments indicating a significant effect on diversity indices and total cover of target plant and co-occurring species in treated as against untreated plots are presented here. For instance, while comparing the response variables of treatment plots with the untreated reference plots after two years of experimentation, we found all treatments significantly increased species richness but it remained almost unchanged in invaded as well as uninvaded reference plots. Species richness increased in all treatment plots with highest richness recorded in *L*. *vulgare* uprooted treatment as indicated by a significant difference (*p* = 0.0001) between treatment and reference invaded plot (Fig 7). This was followed by mowing + herbicide + digging treatment with a significant difference (*p* = 0.001) between treatment and reference invaded plots. Similarly, species evenness and diversity were recorded highest in mowing + herbicide + digging treatment with a significant difference (*p* = 0.001 and *p* = 0.019), respectively between treatment and reference invaded plots (Figs 8 and 9).

**Fig 10:**
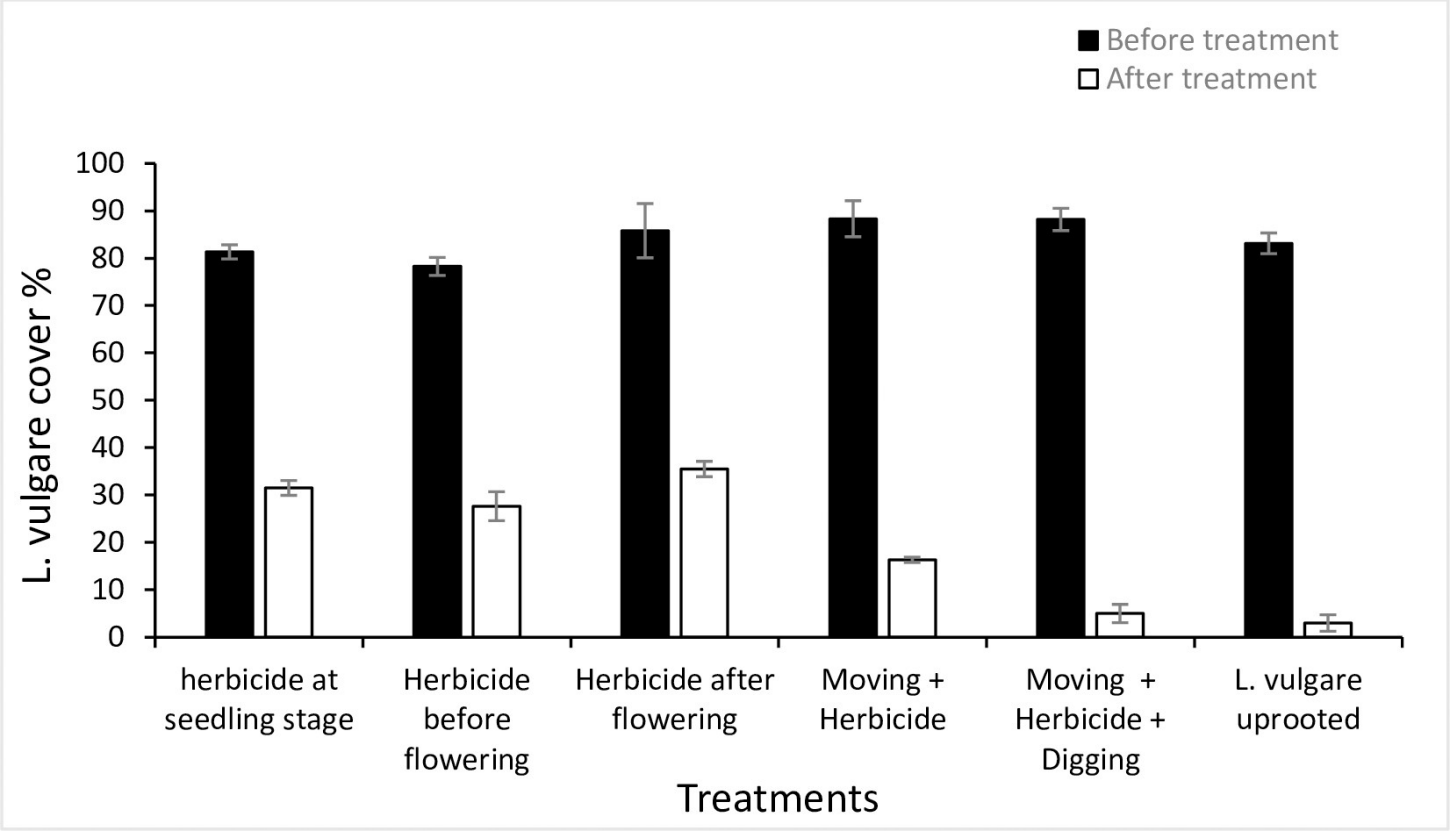
*L*. *vulgare* cover % (per 1 m^2^ plot) before and after treatment for each treatment plot with *L*. *vulgare* uprooted treatment proved to be efficient in reducing the *L*. *vulgare* cover after treatment with a significant difference when compared with reference invaded plot (*p* = 0.0001).

**Fig 11:**
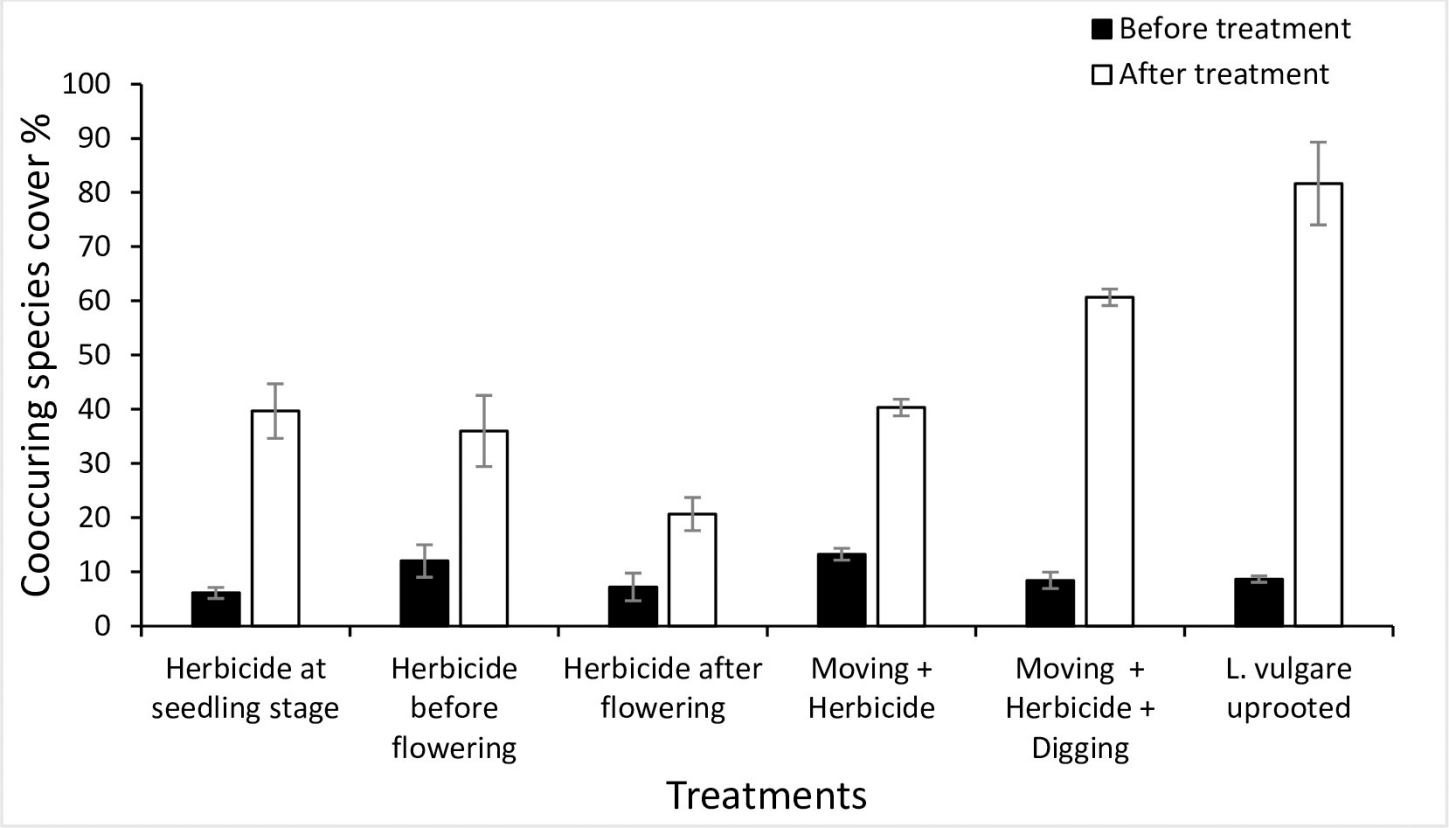
Co-occurring species cover % (per 1 m2 plot) before and after treatment for each treatment plot with *L*. *vulgare* uprooted treatment proved to be efficient in producing highest co-occurring species cover % after treatment with a significant difference when compared with reference invaded plot (p = 0.0001).

In all treatment plots, *L*. *vulgare* cover decreased significantly after 2 years of treatment, whereas for the reference invaded plots the increasing trend was not much significant. Different treatment types differed from each other in their effect on the reduction in the invasive species cover, though maximum reduction in *L*. *vulgare* cover and maximum increase in co-occurring species cover was found in *L*. *vulgare* uprooted treatment with a significant difference of (*p* = 0.0001, and *p* = 0.001) in contrast with invaded reference plots, respectively (Figs 10 and 11).

## Discussion

Significant differences in ecological indices across invaded and uninvaded plots in both the sites indicate that invasion by *L*. *vulgare* reduced species diversity by exerting significant negative impact on natural communities through displacement of native species. Doing so, it brings about discrepancy in structure, composition and functioning of the invaded communities resulting in formation of its large apparent monocultures. This trend is in accordance with a study carried out on this species recently [26] and well corroborated by other studies too [24, 43–45]. The possible reason for the strong negative impact on species diversity by *L*. *vulgare* can be its rapid and vigorous growth and fast reproductive potential [24], that allows it to attain a high cover and form much taller stands as compared to the native co-occurring species in the invaded communities [25]. In apparently looking monoculture stands of *L*. *vulgare* the number of individuals of other species was very minimum. The reproductive success of *L*. *vulgare* as an invasive species may be amplified by its ability to exert competitive influence via allelopathy as five potential allelopathic compounds, such as caprolactam, nonanoic acid, dihydroactinidiolide, dibutyl phthalate, and tetracosane, were isolated recently from this species [46]. Allelopathy can also be attributed to the negative impacts of *L*. *vulgare* on species diversity as these volatile oil containing compounds with herbicidal properties in the flowering parts of this plant can lead to its successful invasion [47].

**Fig 12:**
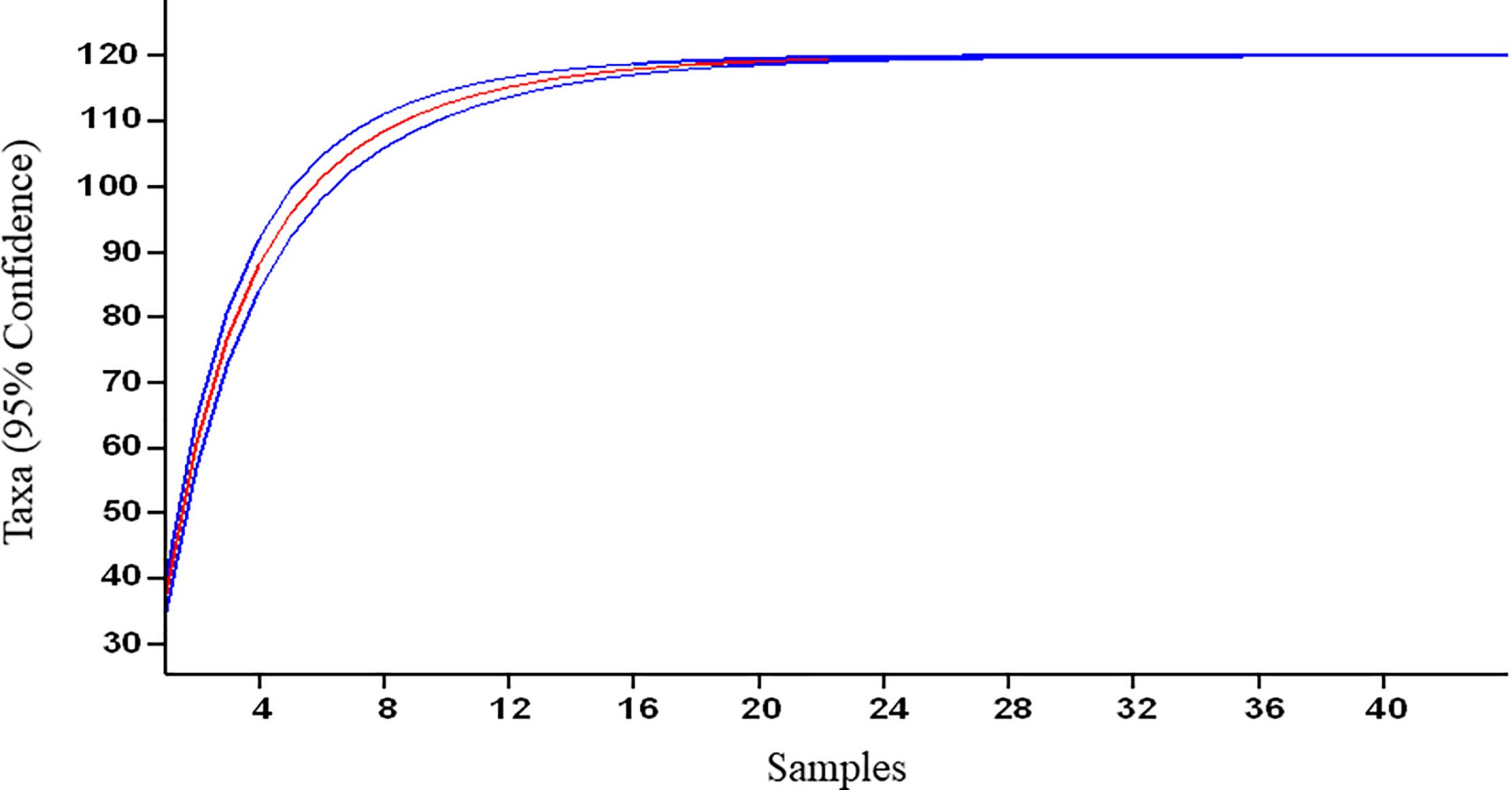
Rarefaction curve showing cumulative number of species recorded as a function of sampling effort.

*L*. *vulgare* is quite predominant in higher reaches and more prevalent in a particular stretch of the Gulmarg tourist spot [26]. To assess sampling completeness, rarefaction curves plotting cumulative number of species as a function of sampling effort were used which indicated that sampling was reasonably complete (Fig 12). The ordination (nMDS) and ANOSIM showed significant differences between species assemblages of invaded and uninvaded plots. The difference was significant for both study sites but the greatest dissimilarity between invaded and uninvaded plots were noticed in Gulmarg. This study is in agreement with the previous study [26] where the similarity percentages (SIMPER) test showed an overall compositional dissimilarity of 65.16% between invaded and uninvaded plots. SIMPER analysis showed dominance of fewer species in invaded plots than in uninvaded plots. These are *Poa annua*, *Cynodon dactylon* and *Fragaria nubicola*.

Plant species diversity increases local net primary productivity through more exploitation of resources as revealed by many diversity-productivity experiments [16]. Interestingly our results indicate that plant invasion results in increase in net primary productivity but decreases local species diversity, which is in agreement with a study [14] where plant invasion is associated with dramatic increase in the net primary productivity as compared to uninvaded communities. The potential explanation for increased net primary productivity in invaded communities is that plant invasion is often associated with increased nutrient pulse, especially nitrogen concentration and nitrification potential in the soil [14, 48] with Ammonia Oxidizing Bacteria (AOB) are quite pertinent drivers of increased nitrification and soil NO_3_ [14]. This is supported by the study [26], where *L*. *vulgare* invaded plots had higher soil nitrogen concentration as compared to uninvaded plots. Other study [49] also reported that *L*. *vulgare* exhibited the best ability to utilize available resources, resulting in significantly greater biomass than the other species tested under the same conditions. Increased productivity of invaders may also be due to lower predation or disease rates [50] and interestingly *L*. *vulgare* is generally avoided by grazing cattle [51].

After being introduced to Kashmir *L*. *vulgare* is now widespread here, especially in the picturesque Gulmarg region. Based on the climatic and biological requirements of the weed from the literature, the forecast is bleak, with possibly forest areas and alpine zones being quite vulnerable to invasion unless a strategy is formulated and implemented soon. Hence, assessment of effectiveness of different types of treatments for ecological restoration of invaded habitats is imperative. Coincidentally, all treatments we tried significantly increased species richness of the invaded habitats. Our results indicate that the restoration treatments used do stimulate the native plant communities as revealed by the variation in species richness and diversity, invasive plant cover and co-occurring species cover between treatment and reference plots.

Herbicide treatment at seedling stage, before flowering and after flowering stages killed all the *L*. *vulgare* plants but many of its new individuals come up again from the rhizomes and soil seed bank. After two years of treatment application, although many of the co-occurring species were seen in the invaded plots but their abundance declined as the cover of *L*. *vulgare* growing from the rhizomes increases. So, these treatments were not efficient enough to eradicate *L*. *vulgare* completely or in recruiting co-occurring species to the invaded plots. Herbicide plus mowing treatment resulted in restoring few co-occurring species but again these were over competed by the *L*. *vulgare* plants that emerged from rhizomes. Overall co-occurring species richness was greater when *L*. *vulgare* was removed with hand-weeding i.e., in *Leucanthemum* uprooting treatment, compared to other treatments. *Leucanthemum* uprooting also proved to be efficient in reducing the *L*. *vulgare* cover and restoring the abundance of co-occurring species in the invaded plots. Manual deweeding is considered better than mechanical deweeding as the later clips the plants from above only and due to over-compensatory growth come up even more vigorously. After *L*. *vulgare* removal, the remaining plants after release of competition show obvious advantage in terms of their growth in cover and abundance. Pertinently, uprooting has been recommended for the control of *L*. *vulgare* by other studies [29]. Manual uprooting also has been seen effective in many other alien invasive species such as *Alliaria petiolata* that can be easily hand pulled [52]. Likewise, removal of invasive forb *Impatiens glandulifera* from riparian areas in the UK resulted in greater species recruitment [53]. A study [54] recommended mowing of *L*. *vulgare* invaded meadows once the first flowers appear, primarily to check the further production and spread of seeds as an effective strategy. Though uprooting will help to prevent seed spread, but it will be difficult to remove the entire rootstock from the ground [55]. The remaining rootstock sprout and new seedlings emerge from the soil seed bank. Thus, follow-up treatments are needed where a persistent seed bank or rhizomes continues to exist. To manage the problem, chemical herbicide treatment seems an option as herbicides such as aminopyralid, metsulfuron, picloram or 2,4-D have been found to temporarily suppress *L*. *vulgare* [29] in meadows. However, the use of herbicides also cannot be the only way to achieve long-term management of *L*. *vulgare* in view of its other environmental implications. Nevertheless, mowing + herbicide + digging treatment, in our case not only suppressed the germination from soil seed bank but proved to be efficient in increasing the species richness, evenness and diversity compared to all other treatments. Our study is supported by fact that mowing stimulates rosette formation [56] which improves the herbicide contact when applied to *L*. *vulgare* [29]. Further support is provided by the studies [57, 58] which suggests that soil loosening by digging remove the standing broom seedlings and seedling germinate from the seed bank. In an experiment in central Alberta provided almost complete control on *L*. *vulgare* by using several types of herbicides such as, Metsulfuron (18 g a.i. ha–1) +2,4-D ester (0.56 kg a.i. ha–1) and Picloram (0.54 kg a.i. ha–1) [55]. Similarly, in western US rangelands the herbicides, such as 2,4-D (2.24 kg a.i. ha–1) and picloram (0.14 kg a.i. ha–1) were found to control *L*. *vulgare* in pastures for 2 to 3 year [56]. Analogously, we used Aminopyralid in combination with 2, 4 D herbicide before flowering, which was quite efficient and effective, in agreement with the earlier studies [59] that recommended use of 2,4-D (2.07 kg a.i. ha–1) and 2,4-D (2.07 kg a.i. ha–1) + clopyralid (30 g a.i. ha–1) for the effective control *L*. *vulgare*. Fertilization at low fertility sites may be appropriate to lessen the re-invasion risk of *L*. *vulgare* [60] by enhancing forage competition, and can effectively eradicate nearly all ox-eye daisy plants [61].

An important outcome from this study is effectiveness of the methods evaluated for restoration indicating a step in the right direction in mitigating the invasion impact. If the most effective treatments are continued for few more years, instead of just two years of treatment, the results promise to be of great value to the managers of such landscapes. The results of this study have an important gap filling value vis-à-vis scientific and practical knowledge in finding the best methods to control invasive plant species. However, the effectiveness of every management plan depends entirely on the leadership of the organization responsible for taking action, its ability to support the funding required, the implementation of a management plan and the involvement and commitment of the authority such as Gulmarg Development Authority, Government of Jammu and Kashmir in this case. Any delay in the management of alien invasive species raises the management costs and reduces the possibility of eradication, as these invasive species do spread rapidly and take over the landscapes. Therefore, preventing the introduction and monitoring the presence and expansion of alien invasive species is the most effective way to safeguard and manage ecosystems from their negative impacts.

## Conclusion

*Leucanthemum vulgare*, a high altitudinal invasive species, has a huge impact on endemic species found in subalpine mountainous ecosystems across globe. It forms dense and quite extensive populations in pastures, which are generally avoided by grazing cattle and even other herbivores. This species is relatively ineffective in preventing soil erosion because of its shallow root system. It also inhibits grass regeneration due to its allelopathic nature. Present study revealed that *L*. *vulgare* invasion reduces native plant diversity to the point where it appears the only plant species present in the invaded habitats in the form of rather monotonous stands. However, decrease in ecological diversity indices in invaded, as compared to the control plots, is associated with concomitant increase in net primary productivity. The increased net primary productivity by invasive plants is generally attribute to their high nitrification potential in the soil, ability to access and use resources more efficiently than the native plant, and a strategy to generally avoid the grazers and herbivores. This is more alarming in view of its impacts on ecosystem processes and functions. Given the impact of *L*. *vulgare* invasion on ecosystem functions, out of the multiple approaches for its management attempted, uprooting before it blossoms and produces seeds seems to be an important strategy. But it must be continued until the soil seed bank is depleted. Another easy way to control *L*. *vulgare* is combined digging and mowing of the plants followed by treatment with herbicides such as Aminopyralid in combination with 2, 4 D. This checks the emergence of new plants from the seed bank and rhizomes as well. To completely eradicate *L*. *vulgare* these approaches, however, need to be continued for some years. Nevertheless, there is need for further research to examine in detail the effects of selective herbicides on native vegetation. As a special case a very targeted application of selected herbicides, especially on completely large monoculture stands of *L*. *vulgare* only, could be considered in combination with other approaches. In the long run the best approach, however, should be avoiding the use of chemical herbicides and instead encourage and upscale the alternative approaches. Though we did not explore the option of biological control, association of *L*. *vulgare* with many insects, viruses, and pathogens in native range [28] indicates the possibility of this option quite a great deal.

## Supporting information

S1 File (XLSX)

S2 File (DOCX)

S1 Technical abstract (PDF)

## References

[1] Foxcroft LC, Pyšek P, Richardson DM, Genovesi P, MacFadyen S. Plant invasion science in protected areas: progress and priorities. Biological Invasions. 2017; 19(5):1353–1378.

[2] Tan PY, Zhang J, Masoudi M, Alemu JB, Edwards PJ, Grêt-Regamey A, et al. A conceptual framework to untangle the concept of urban ecosystem services. Landscape and urban planning, 200. 2020;103837. 10.1016/j.landurbplan.2020.103837 32341614 PMC7183943

[3] Early R, Bradley BA, Dukes JS, Lawler JJ, Olden JD, Blumenthal DM, et al. Global threats from invasive alien species in the twenty-first century and national response capacities. Nature communications. 2016;7(1):1–9. 10.1038/ncomms12485 27549569 PMC4996970

[4] Pyšek P, Jarošík V, Hulme PE, Pergl J, Hejda M, Schaffner U, et al. A global assessment of invasive plant impacts on resident species, communities and ecosystems: the interaction of impact measures, invading species’ traits and environment. Global Change Biology. 2012; 18(5):1725–1737.

[5] Barney JN, Tekiela DR, Barrios‐Garcia MN, Dimarco RD, Hufbauer RA, Leipzig‐Scott P, et al. Global Invader Impact Network (GIIN): toward standardized evaluation of the ecological impacts of invasive plants. Ecology and Evolution. 2015; 5(14):2878–2889. 10.1002/ece3.1551 26306173 PMC4541992

[6] Van Kleunen M, Dawson W, Essl F, Pergl J, Winter M, Weber E, et al. Global exchange and accumulation of non-native plants. Nature. 2015; 525(7567):100–103. 10.1038/nature14910 26287466

[7] Vilà M, Hulme PE. Non-native species, ecosystem services, and human well-being. In *Impact of Biological Invasions on Ecosystem Services*. 2017;1–14. Springer, Cham.

[8] Hejda M, Hanzelka J, Kadlec T, Štrobl M, Pyšek P, Reif J. Impacts of an invasive tree across trophic levels: species richness, community composition and resident species’ traits. Diversity and Distributions. 2017; 23(9):997–1007.

[9] Gaertner M, Den Breeyen A, Hui C, Richardson DM. Impacts of alien plant invasions on species richness in Mediterranean-type ecosystems: a meta-analysis. Progress in Physical Geography. 2009; 33(3):319–338.

[10] Powell KI, Chase JM, Knight TM. A synthesis of plant invasion effects on biodiversity across spatial scales. American Journal of Botany, 98(3), 539–548. 10.3732/ajb.1000402 21613145

[11] Vila M, Ibáñez I. Plant invasions in the landscape. Landscape ecology. 2011; 26(4):461–472.

[12] Ehrenfeld JG. Effects of exotic plant invasions on soil nutrient cycling processes. Ecosystems. 2003; 6(6):503–523.

[13] Liao C, Peng R, Luo Y, Zhou X, Wu X, Fang C, et al. Altered ecosystem carbon and nitrogen cycles by plant invasion: a meta‐analysis. New phytologist. 2008; 177(3):706–714. 10.1111/j.1469-8137.2007.02290.x 18042198

[14] McLeod ML, Cleveland CC, Lekberg Y, Maron JL, Philippot L, Bru D, et al. Exotic invasive plants increase productivity, abundance of ammonia‐oxidizing bacteria and nitrogen availability in intermountain grasslands. Journal of Ecology. 2016; 104(4):994–1002.

[15] Hawkes CV, Wren IF, Herman DJ, Firestone MK. Plant invasion alters nitrogen cycling by modifying the soil nitrifying community. Ecology letters. 2005; 8(9):976–985. 10.1111/j.1461-0248.2005.00802.x 34517683

[16] Hooper DU, Chapin Iii FS, Ewel JJ, Hector A, Inchausti P, Lavorel S, et al. Effects of biodiversity on ecosystem functioning: a consensus of current knowledge. Ecological monographs. 2005;75(1):3–35.

[17] Vanderhoeven S, Dassonville N, Meerts P. Increased topsoil mineral nutrient concentrations under exotic invasive plants in Belgium. Plant and soil. 2005; 275(1–2):169–179.

[18] Ridenour WM, Callaway RM. The relative importance of allelopathy in interference: the effects of an invasive weed on a native bunchgrass. Oecologia. 2001; 126(3):444–450. 10.1007/s004420000533 28547460

[19] Rout ME, Callaway RM. An invasive plant paradox. Science. 2009;324(5928):734–5. 10.1126/science.1173651 19423809

[20] Holmes PM, Esler KJ, Gaertner M, Geerts S, Hall SA, Nsikani MM, et al. Biological invasions and ecological restoration in South Africa. In Biological Invasions in South Africa. 2020; 665–700 Springer, Cham.

[21] Dar GH, Khuroo AA. An introduction to biodiversity of the Himalaya: Jammu and Kashmir state. In *Biodiversity of the Himalaya*: *Jammu and Kashmir State*. 2020; 3–26. Springer, Singapore.

[22] McDougall KL, Alexander JM, Haider S, Pauchard A, Walsh NG, Kueffer C. 2011. Alien flora of mountains: Global comparisons for the development of local preventive measures against plant invasions. Diversity and Distributions 17: 103–111.

[23] Alexander JM, Kueffer C, Daehler CC, Edwards PJ, Pauchard A, Seipel T, MIREN consortium. 2011. Assembly of non-native floras along elevational gradients explained by directional ecological filtering. Proceedings of the National Academy of Sciences 108:656–661. 10.1073/pnas.1013136108 PMC3021079 21187380

[24] Khuroo AA, Malik AH, Reshi ZA, Dar GH. From ornamental to detrimental: plant invasion of *Leucanthemum vulgare* Lam. (Ox-eye Daisy) in Kashmir valley, India. Current Science. 2010; 98(5):600–602.

[25] McDougall K, Wright G, Peach E. Coming to terms with Ox‐eye Daisy (*Leucanthemum vulgare*) in Kosciuszko National Park, New South Wales. Ecological Management & Restoration. 2018; 19(1):4–13.

[26] Ahmad R, Khuroo AA, Hamid M, Malik AH, Rashid I. Scale and season determine the magnitude of invasion impacts on plant communities. Flora. 2019; 260:151481

[27] Benson J. Ox-Eye Daisy: An expanding weed on the tablelands. Nature NSW. 2012; 56:24–25.

[28] Stutz S, Štajerová K, Hinz HL, Müller-Schärer H, Schaffner U. Can enemy release explain the invasion success of the diploid *Leucanthemum vulgare* in North America? Biological Invasions. 2016; 8(7):2077–2091.

[29] Jacobs J. *Ecology and management of oxeye daisy (Leucanthemum vulgare Lam*.*)*. US Department of Agriculture, Natural Resources Conservation Service. 2008.

[30] Polunin O, Stainton A. *Flowers of the Himalaya*. Oxford University Press. 1984.

[31] Stainton A, Polunin O. *Flowers of the Himalaya*. 1988; Oxford University Press.

[32] Ravindranath NH, Ostwald M. Carbon inventory methods: handbook for greenhouse gas inventory, carbon mitigation and roundwood production projects. Springer Science & Business Media; 2007.

[33] Clements DR, Cole DE, King J, McClay A. The biology of Canadian weeds. 128. *Leucanthemum vulgare* Lam. Canadian journal of plant science. 2004; 84(1):343–363.

[34] Magurran AE. Measuring richness and evenness. Trends in ecology & evolution. 1998; 13(4):165–166. 10.1016/s0169-5347(97)01290-1 21238245

[35] Qureshi H, Arshad M, Bibi Y, Osunkoya OO, Adkins SW. Multivariate impact analysis of *Parthenium hysterophorus* invasion on above-ground plant diversity of Pothwar region of Pakistan. Applied Ecology and Environmental Research. 2018; 16(5):5799–5813.

[36] McCune B, Grace JB, Urban DL. *Analysis of ecological communities* (Vol. 28). 2002; Gleneden Beach, OR: MjM software design.

[37] Legendre P, Legendre LF. Numerical ecology. 2012; Elsevier.

[38] Clarke KR, Warwick RM. A further biodiversity index applicable to species lists: variation in taxonomic distinctness. Marine ecology Progress series. 2001; 216:265–278.

[39] Qureshi H, Anwar T, Arshad M, Osunkoya OO, Adkins SW. Impacts of *Xanthium strumarium* L. invasion on vascular plant diversity in Pothwar Region (Pakistan). Annali di Botanica. 2019; 9:73–82.

[40] Clarke KR, Warwick RM. Change in marine communities. *An approach to statistical analysis and interpretation*.2001; 2.

[41] Gooden B, French K, Turner PJ. Invasion and management of a woody plant, *Lantana camara* L., alters vegetation diversity within wet sclerophyll forest in southeastern Australia. Forest Ecology and Management. 2009; 257(3):960–967.

[42] Tererai F, Gaertner M, Jacobs SM, Richardson DM. Eucalyptus invasions in riparian forests: effects on native vegetation community diversity, stand structure and composition. Forest Ecology and Management. 2013; 297:84–93.

[43] Hejda M, Pyšek P, Jarošík V. Impact of invasive plants on the species richness, diversity and composition of invaded communities. Journal of ecology. 2009; 97(3):393–403.

[44] Timsina B, Shrestha BB, Rokaya MB, Münzbergová Z. Impact of *Parthenium hysterophorus* L. invasion on plant species composition and soil properties of grassland communities in Nepal. *Flora-Morphology*, *Distribution*. Functional Ecology of Plants. 2011; 206(3):233–240.

[45] Afreen T, Srivastava P, Singh H, Singh JS. Effect of invasion by *Hyptis suaveolens* on plant diversity and selected soil properties of a constructed tropical grassland. Journal of Plant Ecology. 2018; 11(5):751–760.

[46] Stellick CE, Maddox MM, Quezada S. Investigation of Allelochemicals in *Leucanthemum vulgare*. Metamorphosis. 2020.

[47] Magharri E, Razavi SM, Ghorbani E, Nahar L, Sarker SD. Chemical Composition, Some Allelopathic Aspects, Free-Radical-Scavenging Property and Antifungal Activity of the Volatile Oil of the Flowering Tops of *Leucanthemum vulgare* Lam. Records of Natural Products. 2015; 9(4):538–545.

[48] Lee MR, Flory SL, Phillips RP. Positive feedbacks to growth of an invasive grass through alteration of nitrogen cycling. Oecologia. 2012; 170(2):457–465. 10.1007/s00442-012-2309-9 22526935

[49] Rowland D. Mechanisms of Invasion of *Hieracium aurantiacum* and *Leucanthemum vulgare* in Kosciuszko National Park. 2012.

[50] Keane RM, Crawley MJ. Exotic plant invasions and the enemy release hypothesis. Trends Ecology and Evolution. 2002; 17:164–170. 10.1016/S0169-5347(02)02499-0

[51] Olson BE, Wallander RT. Oxeye daisy (*Chrysanthemum leucanthemum* L.) In: Sheley RL, Petroff JK, editors. Biology and Management of Noxious Rangeland Weeds. Corvallis, Oregon: Oregon State University Press; 1999. 282–9.

[52] Gagnon J. Exotic Invasive Plants. 2020.

[53] Hulme PE, Bremner ET Assessing the impact of *Impatiens glandulifera* on riparian habitats: partitioning diversity components following species removal. Journal of Applied Ecology. 2006; 43(1):43–50.

[54] Georgia AE. *A Manual of Weeds*: *With Descriptions of All the Most Pernicious and Troublesome Plants in the United States and Canada*, *Their Habits of Growth and Distribution*, *with Methods of Control*. 1942; Macmillan.

[55] Cole DE, King JR, Lickacz J. *Integrated control of ox-eye daisy (Chrysanthemum leucanthemum L*.*) in pastures and hayland*. Alberta Agricultural Research Institute. 1999.

[56] Oleson BE and Wallander RT. Oxeye daisy. In: Sheley RL and Petroff JK (eds) Biology and Management of Noxious Rangeland Weeds. Oregon State University Press, Corvallis Oregon. 1999; 282–289.

[57] Parker IM. Haubensak KA, Grove S. Chemical and Mechanical Control of *Cytisus scoparius* Across the Life Cycle. *Technical Report Submitted to Joint Base Lewis-McChord, February 2014*; 36.

[58] Richardson DM, Kluge RL. Seed banks of invasive Australian *Acacia* species in South Africa: role in invasiveness and options for management. Perspectives in Plant Ecology, Evolution and Systematics. 2008; 10(3):161–177.

[59] Sanders P, Rahman A. Evaluation of thifensulfuron for control of some pasture weeds. In *Proceedings of the forty seventh New Zealand plant protection conference, Waitangi, New Zealand, 9–11 August 1994*; 62–67. New Zealand Plant Protection Society.

[60] Mangold J. Invasive Plants and Montana’s Range and Wild Lands. 2017.

[61] Cole DE. Effect of competition on growth of ox-eye daisy (*Chrysanthemum leucanthemum* L.) in pastures and hay land. 1998.

