# Supplementary material for: Ecological restoration of habitats invaded by Leucanthemum vulgare that alters key ecosystem functions
Source: PLoS One. 2021 Mar 26;16(3):e0246665. doi: 10.1371/journal.pone.0246665 (PMC7996977; doi:10.1371/journal.pone.0246665)
Supplement: S2 File — (DOCX) [file pone.0246665.s002.docx]

| **Mean value and standard error of Fig. 6.** | | | | | |
| --- | --- | --- | --- | --- | --- |
| GULMARG | | | DRUNG | | |
| habitat | Mean NPP | Standard Error | habitat | Mean NPP | Standard Error |
| In AGNPP | 1069.12 | 141.6805302 | In AGNPP | 815.4 | 139.470427 |
| Un AGNPP | 358.82 | 17.26705276 | Un AGNPP | 392.4 | 100.9047956 |
| In BGNPP | 309.44 | 5.956882108 | In BGNPP | 203.91 | 37.95569054 |
| Un BGNPP | 151.25 | 9.759610648 | Un BGNPP | 148.41 | 14.57627448 |
|  |  |  |  |  |  |
|  |  |  |  |  |  |
| **Values used to build graph Fig. 7** | | | | |  |
| Diversity-Productivity relationship | | | |  |  |
| site | habitat | NPP gm/m2 | Net pr. productivity | Species richness |  |
| Gulmarg | Invaded | plot 1 | 984 | 11 |  |
| Gulmarg | Invaded | plot 2 | 1272 | 8 |  |
| Gulmarg | Invaded | plot 3 | 1055.2 | 8 |  |
| Gulmarg | Invaded | plot 4 | 1300 | 7 |  |
| Gulmarg | Invaded | plot 5 | 1200 | 8 |  |
| Gulmarg | Invaded | plot 6 | 1300 | 8 |  |
| Gulmarg | Invaded | plot 7 | 1050 | 9 |  |
| Gulmarg | Invaded | plot 8 | 950 | 11 |  |
| Gulmarg | Invaded | plot 9 | 1060 | 11 |  |
| Gulmarg | Invaded | plot 10 | 1320 | 7 |  |
| Drung | Invaded | plot 11 | 950 | 8 |  |
| Drung | Invaded | plot 12 | 754 | 9 |  |
| Drung | Invaded | plot 13 | 910 | 8 |  |
| Drung | Invaded | plot 14 | 915 | 8 |  |
| Drung | Invaded | plot 15 | 720 | 11 |  |
| Drung | Invaded | plot 16 | 950 | 8 |  |
| Drung | Invaded | plot 17 | 825 | 8 |  |
| Drung | Invaded | plot 18 | 945 | 6 |  |
| Drung | Invaded | plot 19 | 940 | 9 |  |
| Drung | Invaded | plot 20 | 845 | 8 |  |
| Gulmarg | Uninvaded | plot 21 | 349.6 | 15 |  |
| Gulmarg | Uninvaded | plot 22 | 369.6 | 16 |  |
| Gulmarg | Uninvaded | plot 23 | 404 | 15 |  |
| Gulmarg | Uninvaded | plot 24 | 400 | 23 |  |
| Gulmarg | Uninvaded | plot 25 | 390 | 21 |  |
| Gulmarg | Uninvaded | plot 26 | 460 | 24 |  |
| Gulmarg | Uninvaded | plot 27 | 455 | 14 |  |
| Gulmarg | Uninvaded | plot 28 | 385 | 14 |  |
| Gulmarg | Uninvaded | plot 29 | 460 | 14 |  |
| Gulmarg | Uninvaded | plot 30 | 415 | 15 |  |
| Drung | Uninvaded | plot 31 | 416 | 11 |  |
| Drung | Uninvaded | plot 32 | 530 | 11 |  |
| Drung | Uninvaded | plot 33 | 306 | 11 |  |
| Drung | Uninvaded | plot 34 | 417 | 12 |  |
| Drung | Uninvaded | plot 35 | 420 | 20 |  |
| Drung | Uninvaded | plot 36 | 390 | 12 |  |
| Drung | Uninvaded | plot 37 | 435 | 9 |  |
| Drung | Uninvaded | plot 38 | 350 | 12 |  |
| Drung | Uninvaded | plot 39 | 340 | 12 |  |
| Drung | Uninvaded | plot 40 | 320 | 14 |  |
|  |  |  |  |  |  |
| **Values used to build graph Fig. 8** | | | | |  |
| Species Richness | Before treatment | St Error | After treatment | St Error |  |
| herbicide at seedling stage | 6.6666667 | 0.5773503 | 9 | 1 |  |
| Herbicide before flowering | 6.6666667 | 0.5773503 | 8 | 1 |  |
| Herbicide after flowering | 7.3333333 | 0.5773503 | 10 | 1 |  |
| Moving + Herbicide | 7 | 1 | 10.666667 | 1.154701 |  |
| Moving + Herbicide + Digging | 6 | 1 | 14 | 1 |  |
| *L. vulgare* uprooted | 7 | 1 | 16 | 2 |  |
|  |  |  |  |  |  |
| **Values used to build graph Fig. 9** | | | | |  |
| Species Evenness | Before treatment | St Error | After treatment | St Error |  |
| herbicide at seedling stage | 0.224 | 0.01053565 | 0.5938 | 0.17327784 |  |
| Herbicide before flowering | 0.30693333 | 0.10988614 | 0.55493 | 0.1919993 |  |
| Herbicide after flowering | 0.2537 | 0.0300045 | 0.40573333 | 0.01149841 |  |
| Moving + Herbicide | 0.25712667 | 0.08886252 | 0.44 | 0.11 |  |
| Moving + Herbicide + Digging | 0.2526 | 0.02997816 | 0.86853333 | 0.09799068 |  |
| L. vulgare uprooted | 0.206 | 0.034906876 | 0.6921 | 0.09012397 |  |
|  |  |  |  |  |  |
| **Values used to build graph Fig. 10** | | | | |  |
| Community diversity | Before treatment | St Error | After treatment | St Error |  |
| herbicide at seedling stage | 0.45973333 | 0.05019724 | 1.224666667 | 0.180112002 |  |
| Herbicide before flowering | 0.62413333 | 0.02961998 | 1.389666667 | 0.09504385 |  |
| Herbicide after flowering | 0.57682667 | 0.01316146 | 1.180333333 | 0.326012781 |  |
| Moving + Herbicide | 0.57626667 | 0.10261415 | 1.658333333 | 0.042524503 |  |
| Moving + Herbicide + Digging | 0.56203333 | 0.01902639 | 2.153333333 | 0.089667906 |  |
| L. vulgare uprooted | 0.45313333 | 0.02719749 | 1.997333333 | 0.306628983 |  |
|  |  |  |  |  |  |
| **Values used to build graph Fig. 11** | | | | |  |
| L. vulgare cover % | Before treatment | St Error | After treatment | St Error |  |
| herbicide at seedling stage | 81.33 | 1.496845 | 31.52 | 1.5632051 |  |
| Herbicide before flowering | 78.263333 | 1.890882 | 27.6666667 | 3.0550505 |  |
| Herbicide after flowering | 85.81 | 5.72443 | 35.5133333 | 1.6221077 |  |
| Moving + Herbicide | 88.333333 | 3.805001 | 16.3333333 | 0.5773503 |  |
| Moving + Herbicide + Digging | 88.18 | 2.347744 | 5 | 1.9403093 |  |
| L. vulgare uprooted | 83.14 | 2.181215 | 3 | 1.7320508 |  |
|  |  |  |  |  |  |
| **Values used to build graph Fig. 12** | | | | |  |
| Cooccurring species cover % | Before treatment | St Error | After treatment | St Error |  |
| Herbicide at seedling stage | 6.11 | 1.0179882 | 39.666667 | 5.033223 |  |
| Herbicide before flowering | 12 | 3 | 36 | 6.557439 |  |
| Herbicide after flowering | 7.22 | 2.5466056 | 20.666667 | 3.05505 |  |
| Moving + Herbicide | 13.2666667 | 1.0863854 | 40.333333 | 1.527525 |  |
| Moving + Herbicide + Digging | 8.4433333 | 1.5032077 | 60.666667 | 1.527525 |  |
| L. vulgare uprooted | 8.6666667 | 0.5773503 | 81.666667 | 7.637626 |  |
