# Supplementary figures and images for: Ecological restoration of habitats invaded by Leucanthemum vulgare that alters key ecosystem functions
Source: PLoS One. 2021 Mar 26;16(3):e0246665. doi: 10.1371/journal.pone.0246665 (PMC7996977; doi:10.1371/journal.pone.0246665)

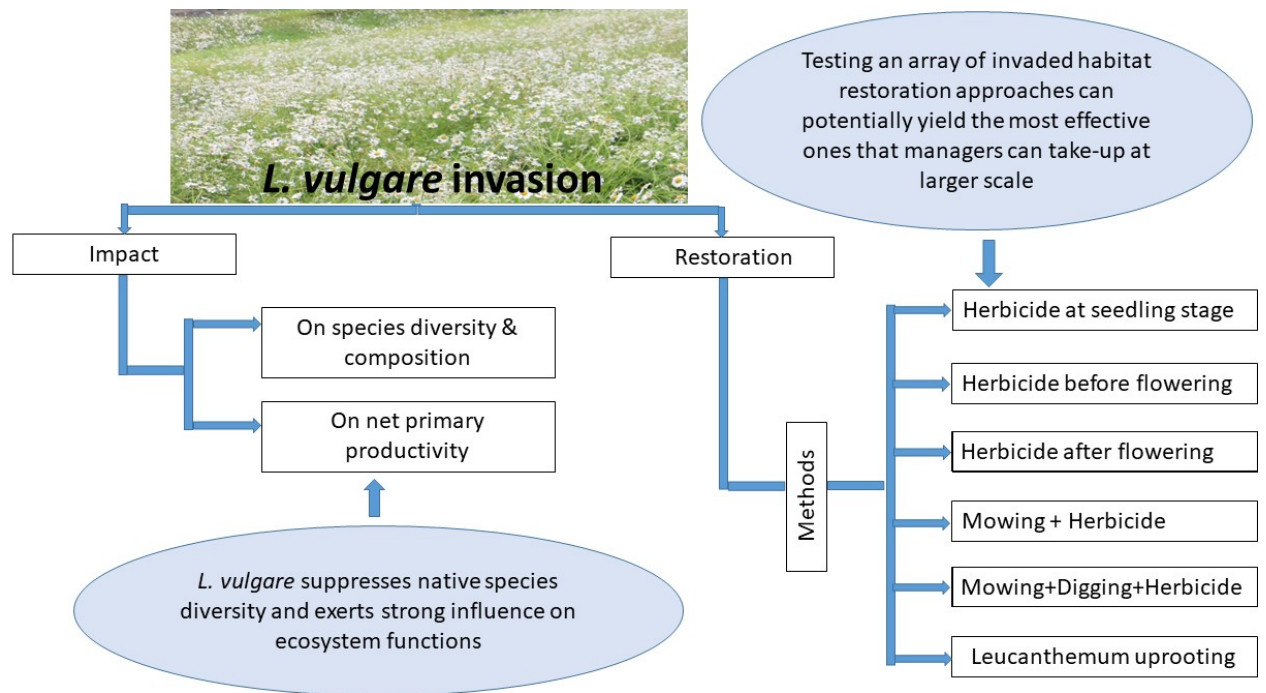

## Technical abstract

Supplement: S1 Technical abstract — (PDF) [file pone.0246665.s003.pdf]
